# Structural and Molecular Mechanism of CdpR Involved in Quorum-Sensing and Bacterial Virulence in *Pseudomonas aeruginosa*

**DOI:** 10.1371/journal.pbio.1002449

**Published:** 2016-04-27

**Authors:** Jingru Zhao, Xiang Yu, Miao Zhu, Huaping Kang, Jinbiao Ma, Min Wu, Jianhua Gan, Xin Deng, Haihua Liang

**Affiliations:** 1 Key Laboratory of Resources Biology and Biotechnology in Western China, Ministry of Education, College of Life Science, Northwest University, Xi’an, ShaanXi, China; 2 Department of Physiology and Biophysics, School of Life Sciences, Fudan University, Shanghai, China; 3 State Key Laboratory of Genetic Engineering, Innovative Collaborative Center of Genetics and Development, Department of Biochemistry, School of Life Sciences, Fudan University, Shanghai, China; 4 Department of Basic Science, School of Medicine and Health Science, University of North Dakota, Grand Forks, North Dakota, United States of America; 5 TEDA Institute of Biological Sciences and Biotechnology, Nankai University, Tianjin, China; Harvard University, UNITED STATES

## Abstract

Although quorum-sensing (QS) systems are important regulators of virulence gene expression in the opportunistic human pathogen *Pseudomonas aeruginosa*, their detailed regulatory mechanisms have not been fully characterized. Here, we show that deletion of PA2588 resulted in increased production of pyocyanin and biofilm, as well as enhanced pathogenicity in a mouse model. To gain insights into the function of PA2588, we performed a ChIP-seq assay and identified 28 targets of PA2588, including the intergenic region between PA2588 and *pqsH*, which encodes the key synthase of *Pseudomonas* quinolone signal (PQS). Though the C-terminal domain was similar to DNA-binding regions of other AraC family members, structural studies revealed that PA2588 has a novel fold at the N-terminal region (NTR), and its C-terminal HTH (helix-turn-helix) domain is also unique in DNA recognition. We also demonstrated that the adaptor protein ClpS, an essential regulator of ATP-dependent protease ClpAP, directly interacted with PA2588 before delivering CdpR to ClpAP for degradation. We named PA2588 as CdpR (ClpAP-degradation and pathogenicity Regulator). Moreover, deletion of *clpP* or *clpS/clpA* promotes bacterial survival in a mouse model of acute pneumonia infection. Taken together, this study uncovered that CdpR is an important QS regulator, which can interact with the ClpAS-P system to regulate the expression of virulence factors and pathogenicity.

## Abstract 2

The novel transcriptional regulator CdpR suppresses the expression of virulence factors and pathogenicity in *Pseudomonas aeruginosa* and is degraded by the ClpAS-P protease system.

## Author Summary

Although many transcriptional regulators tune *P*. *aeruginosa* virulence factor expression and secretion, the molecular mechanisms of the underlying regulatory network are still elusive. Quorum sensing, the ability of bacteria to communicate and detect cell density to determine the most advantageous time to orchestrate collective events, is known to govern *P*. *aeruginosa* virulence. In this study, we present a novel AraC-family transcription factor, CdpR (PA2588), that controls numerous virulence factors via directly regulating the quorum sensing *Pseudomonas* quinolone signal (PQS) system. We solved the crystal structure of CdpR, which showed that its N-terminal domain contains a unique fold that is different from other AraC-family proteins. In addition, we found that CdpR is regulated by the ClpAS-ClpP protease. CdpR interacts with the adaptor protein ClpS and then is degraded by the ATP-dependent ClpAP protease. This is the first example of a quorum-sensing regulator as a substrate of the ClpAS-ClpP protease in *P*. *aeruginosa*. These findings significantly extend our understandings of quorum sensing and virulence regulation and provide insights into the function of proteases in pathogenic bacteria.

## Introduction

*P*. *aeruginosa* is one of the most common nosocomial pathogens associated with fatal lung disease in cystic fibrosis patients [1]. This bacterium synthesizes a group of virulence factors consisting of pyocyanin, rhamnolipids, proteases, and biofilms that are regulated by a cell density-dependent quorum-sensing (QS) system [2,3] and second messenger c-di-GMP [4].

QS systems enable bacteria to communicate and regulate a large number of genes. *P*. *aeruginosa* has one of the most sophisticated QS systems of all bacterial species, which includes two systems (*las* and *rhl*) based on N-acyl-homoserine lactones (AHLs) and one based on 2-alkyl-4-quinolone (AQ) [2]. The LasI synthase produces the signal of the *las* system, *N*-3-oxo-dodecanoyl homoserine lactone (3-oxo-C_12_-HSL), which is recognized by the transcriptional regulator LasR [5]. The RhlI synthase catalyzes the synthesis of *N*-butanoyl homoserine lactone (C_4_-HSL), which is sensed by the transcriptional regulator RhlR [6]. These two systems control 10% of the *P*. *aeruginosa* genome [7,8]. Besides the AHLs systems, *P*. *aeruginosa* also produces the AQ signal molecule [9]. *Pseudomonas* quinolone signal (PQS) is synthesized from the precursor molecule 2-heptyo-4(1H)-quinolone (HHQ) and finally converted into PQS by PqsH [10]. Moreover, these three systems and a group of transcriptional regulators (such as VqsR, QscR, VqsM, Vfr, and RpoN) form a complex regulatory network [11].

Intracellular protein degradation plays essential roles in many physiological processes such as cell growth and division. In *Escherichia coli*, proteolysis is mainly mediated by ATP-dependent proteolytic machineries (termed AAA+ proteases) [12], which consist of a proteolytic barrel (e.g., heptameric ClpP) and a hexameric ATPase (e.g., ClpA, ClpX). The ATP-binding proteins ClpX and ClpA recognize a specific peptide sequence of unfolded proteins [13,14], and translocate the tags into the peptidase barrel of ClpP, thus degrading the unfolded protein into small peptides [15]. In addition, adaptor proteins (i.e., ClpS) are responsible for binding and transporting their target proteins to the protease complexes for degradation [16]. In *P*. *aeruginosa*, the Lon and ClpP proteases share 84% and 86% identity with their *E*. *coli* counterparts, which suggest that these proteases also play important roles in the degradation of unstable or misfolded proteins in *P*. *aeruginosa*. Moreover, the ATP-dependent ClpP protease has been shown to control virulence, antibiotic resistance, and a group of metabolic pathways [17]. However, the substrates and molecular mechanism of ClpP in regulating bacterial pathogenesis are still unclear.

In this study, we showed that the new QS regulator CdpR (we named PA2588 in this study) directly activated PqsH, the PQS synthase. CdpR regulated the expression of virulence factors (i.e., pyocyanin and motility) and bacterial pathogenicity of *P*. *aeruginosa*. Unlike other AraC-family proteins, the C-terminal HTH (helix-turn-helix) motif of CdpR has a unique conformation, which may play important roles in DNA recognition. Structural comparison also revealed that the N-terminal region (NTR) of CdpR has a novel fold whose function remains elusive. We also found that CdpR interacts with adaptor protein ClpS and is degraded by the ClpAP protease in vivo. Moreover, the deletion of *clpP* or *clpS/clpA*-promoted bacterial survival in a mouse model of acute pneumonia. To the best of our knowledge, this is the first report showing the degradation of a regulator of the alginate system by ClpAS-P proteases in *P*. *aeruginosa*. Overall, these findings establish a link between proteases ClpAS-P and virulence, which provides us with new insights into investigate the roles of proteases in the future.

## Results

### Deletion of *cdpR*-Altered *P*. *aeruginosa* Phenotypes

**Fig 1:**
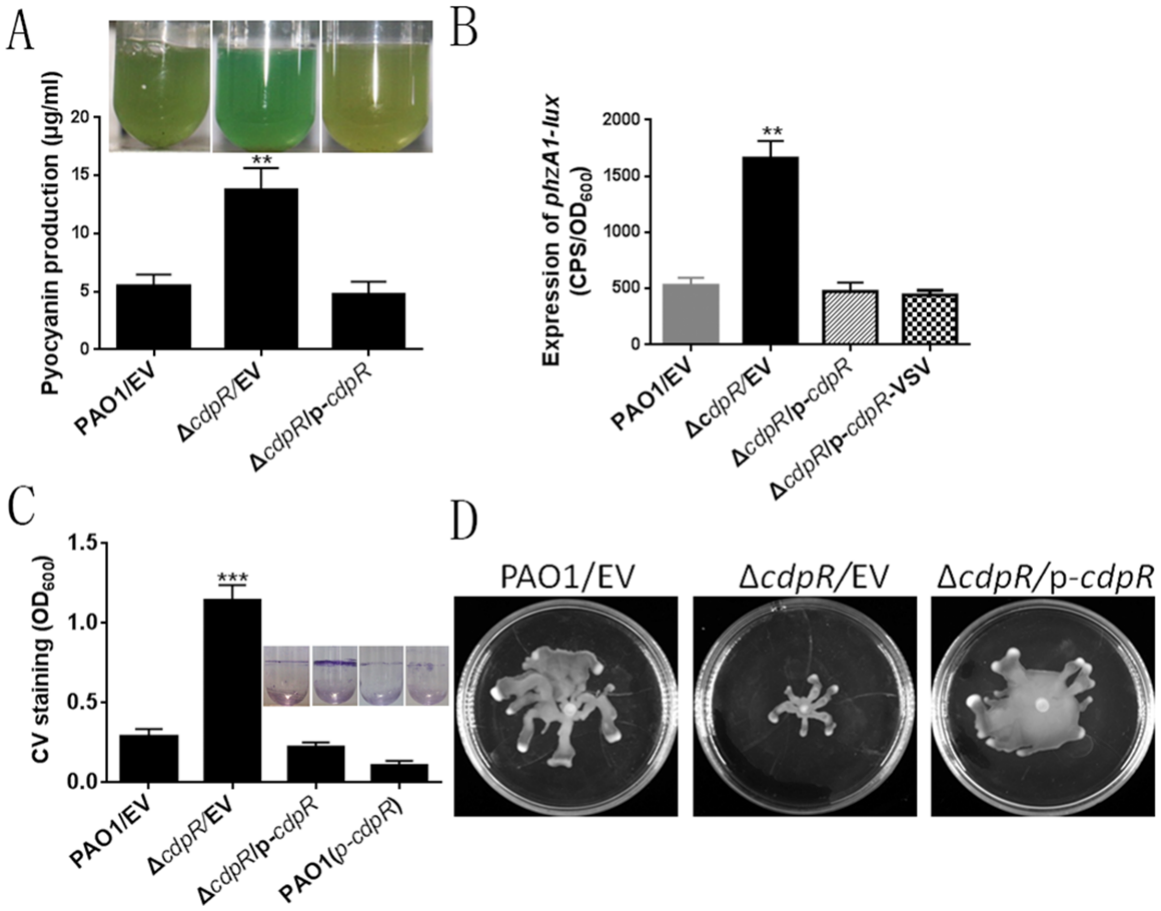
Deletion of the *cdpR* gene in *P*. *aeruginosa* alters numerous phenotypes. **(A)** The production of pyocyanin was measured in wild-type PAO1, Δ*cdpR* mutant, and Δ*cdpR*-complemented (Δ*cdpR/p-cdpR*) strains. The photos show overnight strains cultured in LB broth. The asterisks indicate that the pyocyanin production in Δ*cdpR* strain is statistically different from wild-type strain as determined by a Student’s *t* test (**p < 0.05). EV represents empty vector. **(B)** Expression of *phzA1* was drastically repressed in the Δ*cdpR* mutant compared to that in wild-type and the Δ*cdpR* complemented strains. **p < 0.05 compared to Wild-type (WT) or complemented strains by Student’s *t* test. Data are means ± standard deviation (SD). EV represents empty vector. **(C)** CdpR is required for biofilm formation. Quantification of crystal violet staining of biofilms was performed after growth in microtiter plates for 14 h. ***p < 0.001 compared to WT or complemented strains by Student’s *t* test. A photo of the tube binding assay was taken. EV represents empty vector. **(D)** Effect of *cdpR* mutation on swarming motility. Overnight cultures were spotted onto swarming plates (2 μl aliquots) and the plates were incubated at 37°C. The images captured after 16 h of growth. The experiments were repeated at least three times, and similar results were observed.

Previously, we showed that the QS regulator VqsM directly binds to the promoter region of the *PA2588*(*cdpR*) gene [18]. Its N-terminus (amino acids 59–198) is homologous to the arabinose-binding domain of the AraC transcription regulator (N-term; pfam12625), suggesting that it may be an AraC-family transcriptional regulator. Given that *lasI* and *cdpR* are directly controlled by VqsM, we hypothesized that CdpR also regulates *P*. *aeruginosa* QS-associated virulence factors, such as pyocyanin production. To this end, we made a *cdpR* null mutant in the PAO1 strain by following procedures from our previous study [18]. The resulting Δ*cdpR* mutant exhibited higher pyocyanin production than wild-type PAO1 after 24 h of growth in Luria-Bertani (LB) medium. Overexpression of *cdpR* in a pAK1900 plasmid (T7 promoter) decreased the pyocyanin production in the *cdpR* mutant (Fig 1A).

Two homologous operons are involved in the synthesis of phenazine compounds (i.e, pyocyanin) in *P*. *aeruginosa*, *phzA1B1C1D1G1* (*phzA1*) and *phzA2B2C2D2G2*(*phzA2*) [19]. The increased pyocyanin production in the Δ*cdpR* strain led us to test if *cdpR* regulates the promoter activity of *phzA1*. To this end, a *phzA1-lux* reporter was introduced into the wild-type, the Δ*cdpR* mutant, and its complemented strain. The expression of *phzA1-lux* in the Δ*cdpR* mutant was 3-fold higher than in the wild-type PAO1 or its complemented strain (Fig 1B). These results suggest that CdpR is a negative regulator of pyocyanin synthesis.

The enhanced pyocyanin production in the Δ*cdpR* mutant led us to test if *cdpR* regulates other QS-related pathways, such as biofilm formation and bacterial motility. We performed microtiter dish and borosilicate tube binding assays to evaluate the biofilm formation of these strains. The Δ*cdpR* mutant exhibited better adherence than either the wild-type or the complemented strain (Fig 1C). In addition, deletion of *cdpR* resulted in reduced swarming motility (Fig 1D). Collectively, these results demonstrated that CdpR is a new and important regulator of *P*. *aeruginosa* virulence factors.

### ChIP-seq Identified CdpR Targets in *P*. *aeruginosa* Genome

**Fig 2:**
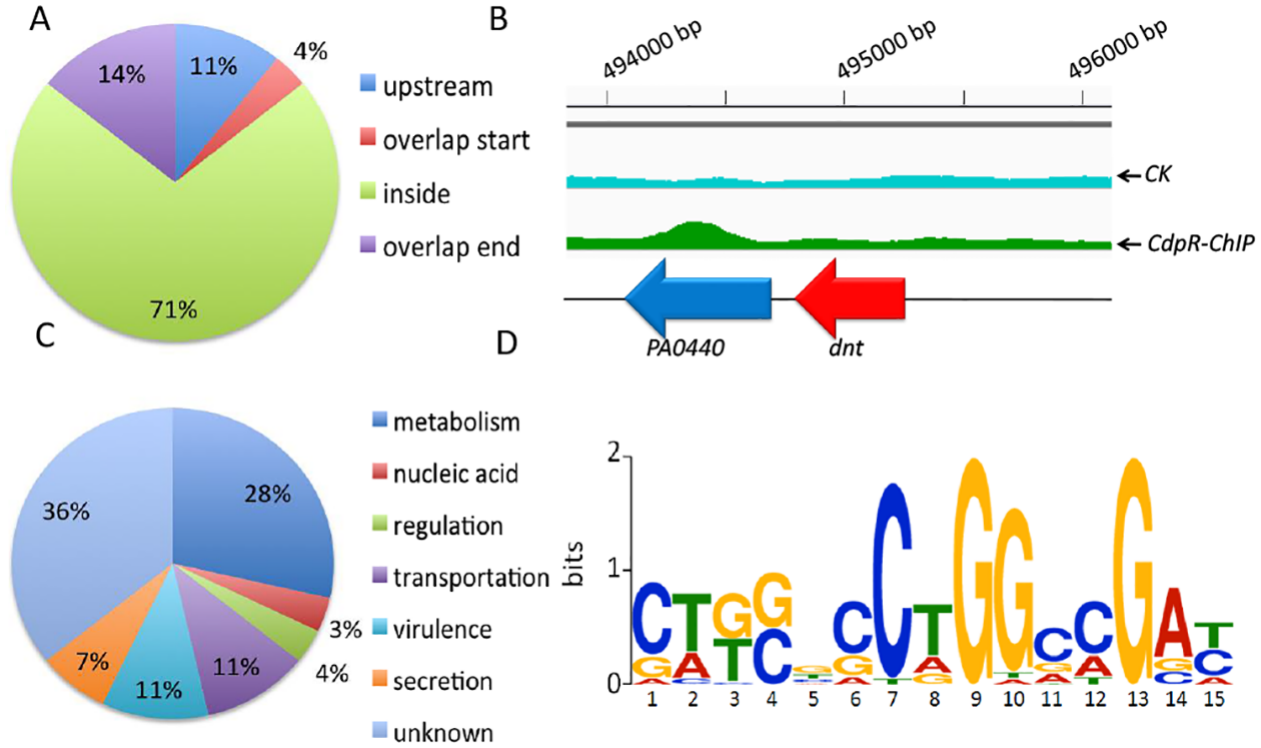
ChIP-seq and RNA-seq identify CdpR regulons in *P*. *aeruginosa* genome. **(A)** The positions of the CdpR-binding peaks are presented in a pie chart. **(B)** CdpR binds to the inside sequence of PA0440. **(C)** Pie chart presents the percentages of CdpR targets with functional categories defined in the *Pseudomonas* database (http://pseudomonas.com). **(D)** The most highly significant motif identified by ChIP-seq using the MEME tool is shown. The height of each letter represents the relative frequency of each base at different position in the consensus sequence.

In order to dissect the regulatory mechanism of CdpR, we performed a ChIP-seq assay to identify its direct targets in the *Pseudomonas* genome. Like untagged CdpR, CdpR-VSV (overexpressed in the plasmid pAK1900) was also able to decrease the expression of *phzA1* (Fig 1B).Using MACS software for identifying conserved DNA motif bound by CdpR [20], 28 enriched loci (*p*-value = e^−5^) were identified to carry CdpR-binding peaks (S1 Table), which were enriched > 1.5 folds compared to the control samples using the wild-type without VSV tags. Eighty-nine percent of these 28 loci are located within coding regions (Fig 2A). For example, CdpR binds to the coding region of PA0440 gene in the immunoprecipitated sample, while the enrichment in PA0440 was not present in the input sample (the wild-type PAO1 strain with empty pAK1900 vector) (Fig 2B). We further categorized the biological processes of CdpR targets based on gene ontology [21], including metabolism (28%), secretion (7%), regulation (4%), nucleic acid metabolism (3%), transportation (11%), virulence (11%), and unknown functions (36%) (Fig 2C). The ChIP-seq data also allowed us to define a 15-bp consensus-binding motif of CdpR (Fig 2D) using multiple EM for motif elicitation (MEME) analysis.

We next sought to perform electrophoretic mobility shift assays (EMSA) to verify the CdpR targets in vitro. We first expressed and purified the CdpR protein (S1A Fig). CdpR bound to two promoter regions of *cerN* and *sphR*, but not to the *lasR* promoter as a negative control (S1B–S1F Fig). We further tested 18 binding sites, and 13 showed specific binding (S2A Fig). Overall, these EMSA results are consistent with the ChIP-seq data and confirm the binding motif of CdpR in the *P*. *aeruginosa* genome.

### CdpR-Regulated Expression of *pqsH* and Itself

**Fig 3:**
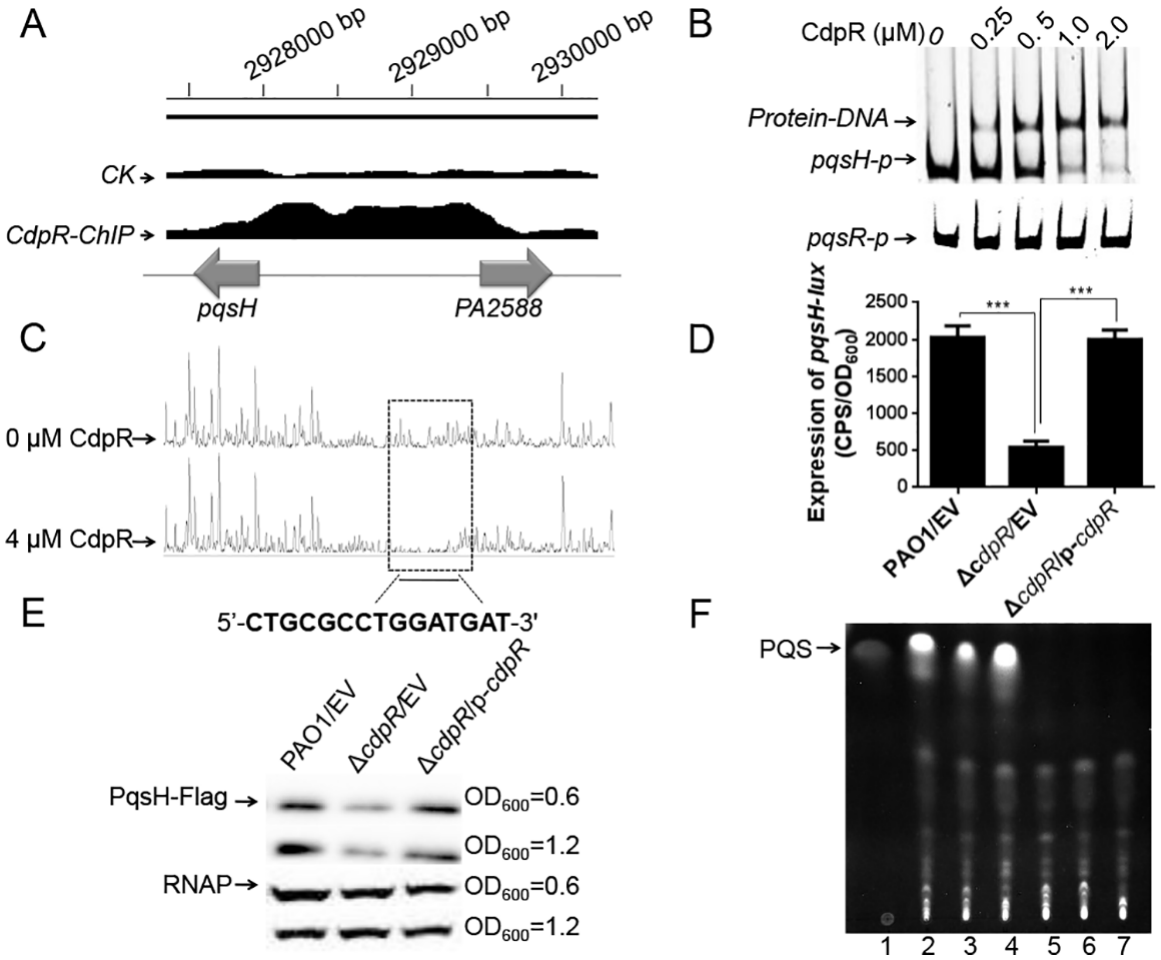
CdpR directly binds to *pqsH-cdpR* intergenic region and regulates *pqsH* expression. **(A)** CdpR bound to the *pqsH*-*cdpR* intergenic region according to the ChIP-seq analyses. **(B)** EMSA experiment showed that CdpR directly bound to the *pqsH*-*cdpR* intergenic region (*pqsH-p*) but not to that of *pqsR*. PCR products containing *pqsH* or *pqsR* promoter regions were added to the reaction mixtures at a concentration of 40 nM. The protein concentration (μM) for each sample is indicated above its lane. **(C)** CdpR bound to the motif (CTGCGCCTGGATGAT) in the *pqsH* promoter region. Electropherograms show the protection pattern of the *pqsH* promoter region after digestion with DNase I following incubation in the absence or presence of 4.0 μM CdpR. The protected region showed significantly reduced peak pattern than seen in the control. **(D)** The activity of *pqsH* was drastically activated by CdpR *in vivo*. The expression of *pqsH* was measured in the wild-type PAO1, the Δ*cdpR* mutant, and the complemented strain (Δ*cdpR*/p-*cdpR*). ***p < 0.001 based on Student’s *t* test. EV represents empty vector. **(E)** Western-blotting confirms that the expression of *pqsH* was drastically lower in the Δ*cdpR* strain than in the wild-type PAO1. The wild-type PAO1, the Δ*cdpR* mutant, and the Δ*cdpR* complemented strain containing the integrated single-copy plasmid CTX-*pqsH*-flag were cultured at OD_600_ = 0.6 and OD_600_ = 1.2. The whole-cell extracts from the designated strains were subjected to SDS/PAGE separation and subsequent immuno-blotting. EV represents empty vector. **(F)** The production of PQS was decreased in the *cdpR* mutant. Lane 1 contains 50 ng of PQS. Lanes 2–7 represent the wild-type PAO1, the Δ*cdpR* mutant, the Δ*cdpR* complemented strain, the Δ*pqsH* mutant, the Δ*pqsR* mutant, and the Δ*pqsA* mutant, respectively. EV represents empty vector.

The highest enriched region by CdpR was the shared promoter region between *pqsH* and *cdpR* (Fig 3A). EMSA showed that CdpR bound to the *pqsH*-*cdpR* intergenic region (*pqsH-p*) but not to the negative control (*pqsR-p*) (Fig 3B). Using a dye-based DNaseI footprinting assay, we further found a specific CdpR-bound sequence containing a 15-bp motif (5'-CTGCGCCTGGATGAT-3') (Fig 3C). The protected region extended from nucleotides -377 to -361 ahead of the *pqsH* translational start codon, which is consistent with the identified sequences by MEME analysis (Fig 2D). We have blasted the intergenic sequences between *cdpR* and *pqsH* in other *Pseudomonas* species. This region is largely conserved in *P*. *aeruginosa*, but not in other *Pseudomonas* species, suggesting that the shared promoter between *cdpR* and *pqsH* is universal in *P*. *aeruginosa*. We also noted that the motif (CTGCGCCTGGATGAT) is also present in the promoter regions of *cerN* (5'-CTTCGCCTGGTTGCC-3', -87 to -72 from the *cerN* translational start codon) and *sphR* (5'-CTGCGCCTGGCCGCC-3', -266 to -252 from the *sphR* translational start codon) (S2B Fig).

In order to determine if the motif (CTGCGCCTGGATGAT) was important for binding to CdpR, we performed EMSAs again using a *pqsH-p1* DNA fragment (−365 to +88 relative to the start codon of *pqsH*, without the binding motif) and a *pqsH-M-p* DNA fragment (the motif CTGCGCCTGGATGAT was mutated to TGACTTCTGGATGAT). Neither the *pqsH-p1* nor *pqsH-M-p* fragments were able to bind to CdpR (S3A Fig). As expected, CdpR could not bind to the *sphR-p1* or *cerN-p1* fragment (without the binding motif) (S1D and S1E Fig).

We next tested if CdpR controls the expression of *pqsH* or itself in vivo. As shown in Fig 3D, the promoter activity of *pqsH* (*pqsH-lux*, −500 to +88 relative to the start codon) in the Δ*cdpR* mutant strain was drastically weaker than that in the parental strain. Overexpression of *cdpR* gene in the Δ*cdpR* strain restored the expression of *pqsH* to the wild-type level. This result was further echoed by western blotting of the strains carrying an integrated mini-CTX-*pqsH*-*flag* plasmid (Fig 3E). We further measured QS molecules including PQS and C_4_-HSL, which showed that PQS production was lower in the *cdpR* mutant than in the wild-type PAO1 strain (Fig 3F); but no difference in C_4_-HSL production was observed between them (S3B Fig). Moreover, mutation in the consensus motif abolished the promoter activity of *pqsH-M-lux* in the wild-type strain (S3C Fig), suggesting that the binding sequence is required for full activity of the *pqsH* promoter. In addition, the expression of *cdpR* in the Δ*cdpR* mutant was 4-fold higher than that in the wild-type strain (S3D Fig), suggesting that the *cdpR* is negatively autoregulated. Taken together, these results indicate that CdpR regulates both *pqsH* and itself.

### Deletion of *cdpR* Resulted in Increased Bacterial Pathogenesis in a Mouse Model

**Fig 4:**
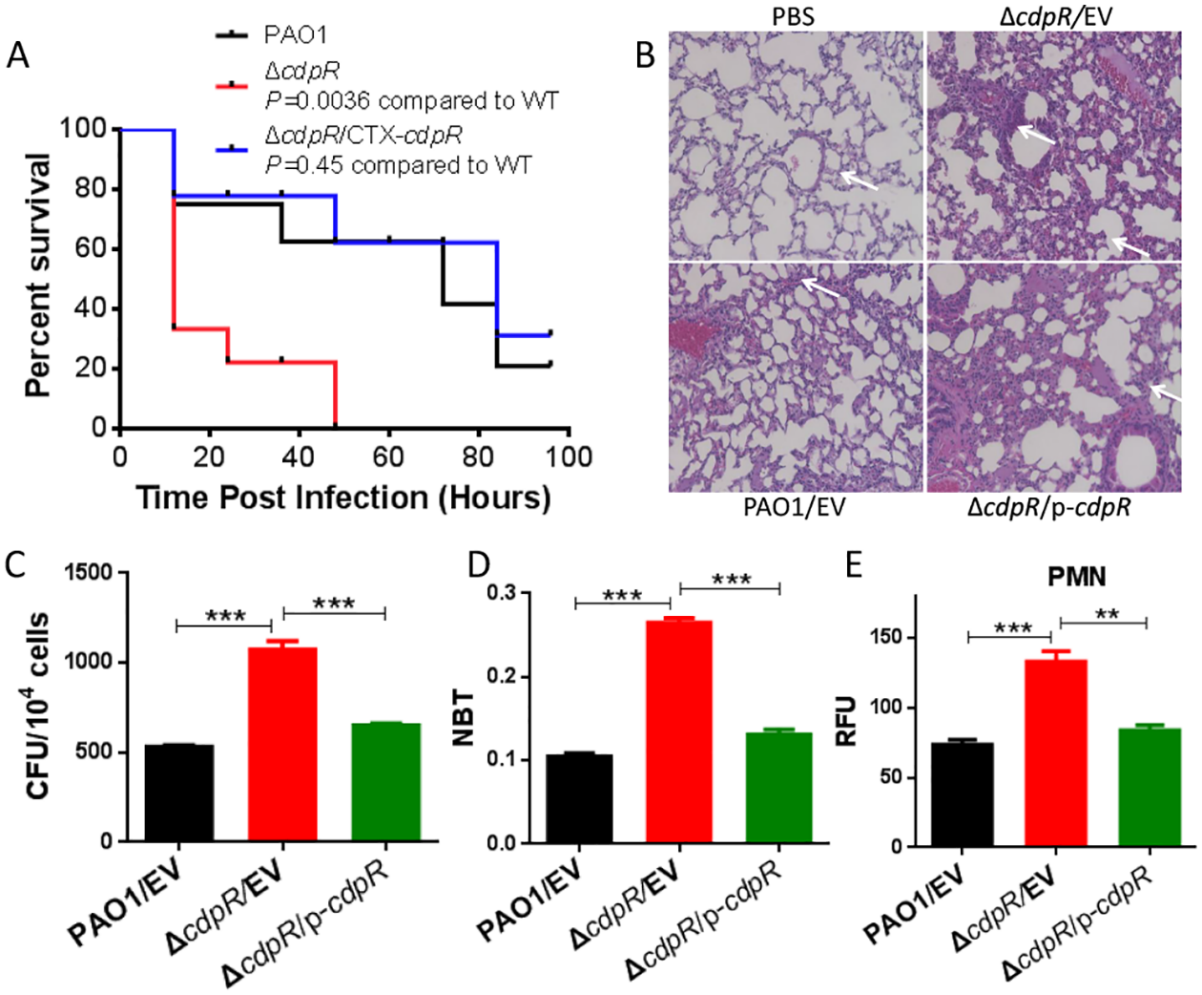
**(A)** The *cdpR* deletion increased the virulence of *P*. *aeruginosa*. C57BL6 mice were intranasally challenged with Δ*cdpR*, Δ*cdpR* complemented strain, and wild-type PAO1 at 1 × 10^7^ cfu in 50 μl phosphate buffered saline (PBS), moribund mice were killed to obtain survival data (Kaplan-Meier Curve with Log-Rank test, *p* = 0.0036, *n* = 6). **(B)** The *cdpR* deletion mutant increased lung injury and inflammatory response in mice (indicated by arrows). C57BL6 mice were intranasally challenged with the Δ*cdpR* strain, the complemented strain, and wild-type PAO1 at 10^7^ cfu in 50 μl PBS, and mice were killed for histological analysis 48 h after infection (H&E stain, original magnification × 400). Data were representative of six mice. **(C)** Bacterial burdens of AMs of bronchoalveolar lavage (BAL) were detected by CFU assay. 5,000 AM of each mouse were incubated in a LB-agar dish at 37°C overnight. **(D)** Superoxide levels were determined by a Nitroblue Tetrazolium (NBT) assay. Data were mean ± SEM and representative of three experiments. **(E)** PMN infiltration in blood was counted by Hema staining. **p < 0.05 or ***p < 0.001 based on Student’s *t* test. EV represents empty vector.

As aforementioned, the deletion of *cdpR* increased pyocyanin production and compromised the swarming motility and improved biofilm formation (Fig 1). We next sought to test whether the deletion of *cdpR* changes *P*. *aeruginosa* virulence in a mouse model of acute pneumonia. Therefore, 6-wk-old C57BL/6 mice were intranasally inoculated with 1×10^7^ wild-type, Δ*cdpR* mutant, or complemented strain (single copy integration of the *cdpR* gene). Kaplan-Meier survival analysis showed that loss of *cdpR* significantly decreased mouse survival compared to the wild-type PAO1. The Δ*cdpR* strain caused 70% and 100% of infected mice to die by 12 h and 48 h, respectively. In contrast, 60% of mice infected with wild-type PAO1 or the complemented strain were alive at 48 h (Fig 4A). We also observed more lung injury and inflammation in the Δ*cdpR* strain-infected mice than the wild-type PAO1 or complemented strain-infected mice by histological analysis with H&E staining (Fig 4B). The colony-forming units (CFUs) in alveolar macrophages (AM) infected with Δ*cdpR* were higher than those with the wild-type or the complemented strain (Fig 4C). In addition, the Δ*cdpR*-infected mice produced more superoxide and more polymorphonuclear neutrophil (PMN) cells than wild-type-infected mice (Fig 4D and 4E). In summary, these results indicate that CdpR is a negative regulator of *P*. *aeruginosa* pathogenicity in a mouse model.

### Overall Structure of CdpR and Novel NTR Folding

**Fig 5:**
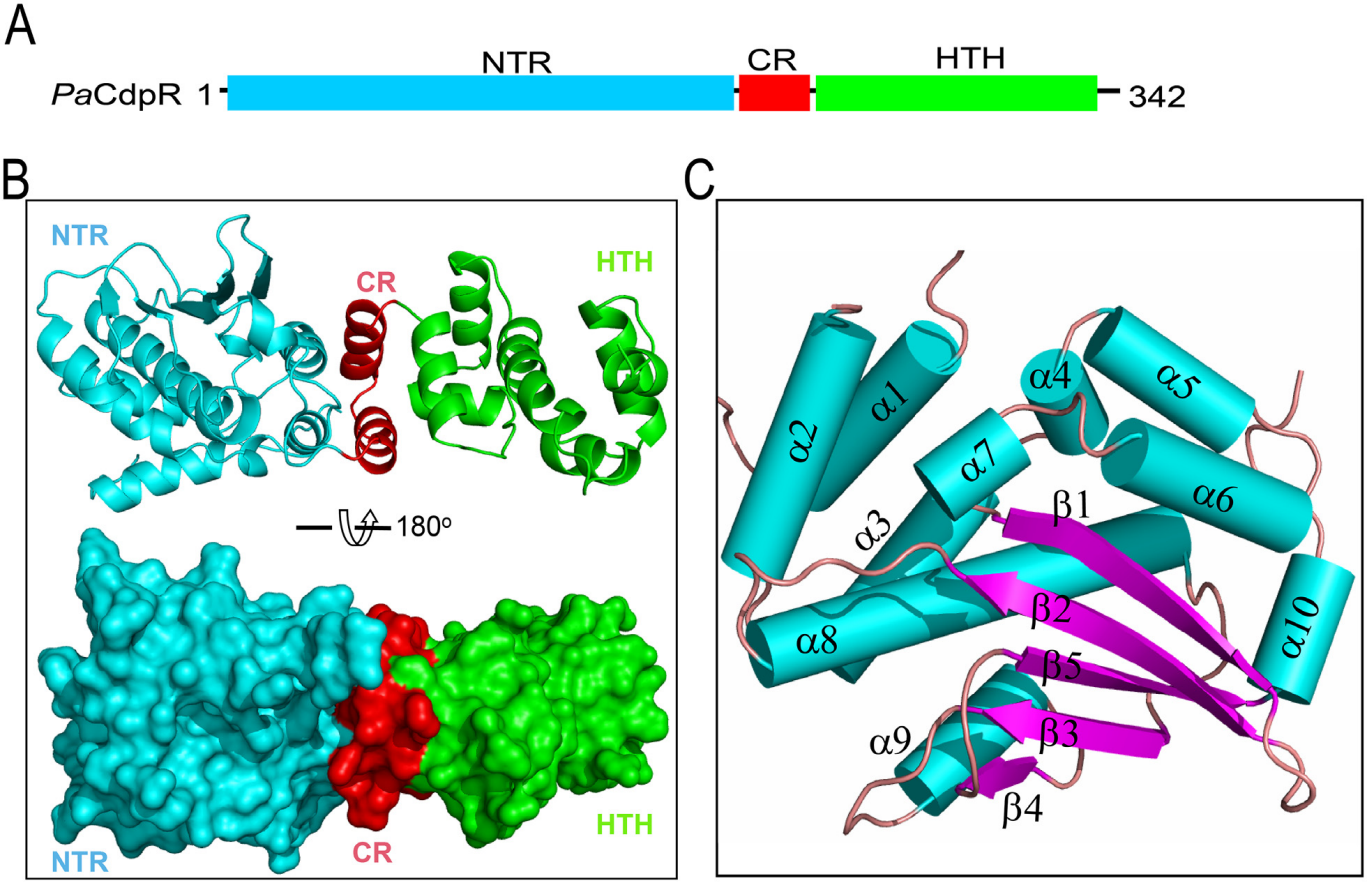
The overall structure of *Pa*CdpR. **(A)** Schematic diagram of the domain architecture of *Pa*CdpR. **(B)** Cartoon and surface presentation showing the overall structure of *Pa*CdpR and the assembly of NTR, CR, and HTH domains, which are colored in cyan, red, and green, respectively. **(C)** The overall fold of NTR. The structure was colored according to the secondary structures (α-helices, cyan; β-strand: magenta; loop: pink).

To gain more insights into the structural basis underlying the functions of *Pa*CdpR, we carried out structural studies. The structure belongs to P4_1_2_1_2 space group, and it contains one *Pa*CdpR protein molecule in the asymmetric unit. As depicted in Fig 5A and 5B, the overall structure of *Pa*CdpR can be divided into three regions, i.e., NTR (aa 5–203), connector region (CR, aa 204–228), and C-terminal HTH domain (aa 229–329). The NTR (Fig 5C) is of *Pa*CdpR that is of α/β fold in nature; it is composed of ten α-helices (α1 to α10) and five β-strands (β1 to β5). The β strands form a single-layer β-sheet. The last strand (β5) sits in the center, whereas β2 and β1 locate on one side, and β3 and β4 locate on the other side. Except β4, which is parallel to β3, all the other strands are antiparallel to each other. The α-helices can be divided in two groups: group A (α1 to α5) and group B (α6 to α10). Group B α-helices were clamped between the A group helices and the β-sheet. Four helices, α9, α10, α6, and α7 were packed against the β-sheet at the edge in an anti-clockwise direction, while α8 (the longest helices) crossed the β-sheet in the middle and formed extensive hydrophobic interactions with the three central strands (β2, β5, and β3).

*E*. *coli* AraC (*Ec*AraC) is composed of two domains, i.e., N-terminal domain (NTD) and C-terminal HTH domain, and is the most well studied member of AraC family. Upon the binding of arabinose, *Ec*AraC NTD can change its dimerization mode, which will in turn alter the HTH–DNA interaction and affect its gene regulation activity [22]. Though both CdpR NTR and *Ec*AraC NTD are of α/β fold in nature, their overall folds are different from each other. *Ec*AraC NTD has a jelly-roll fold β-barrel, which holds the arabinose in between the double-layer beta-sheet (S4A Fig). Moreover, no sequence similarity was observed between CdpR NTR and *Ec*AraC NTD. These differences suggested that CdpR NTR and *Ec*AraC NTD may have different functions. To this end, we carried out an in vitro DNA binding assay. As shown in S4B Fig, the CdpR–DNA interaction was almost identical in the presence or absence of arabinose, suggesting that arabinose is not a cofactor of CdpR. Structural comparison using DALI server [23] did not reveal significant similarity between CdpR NTR and other known AraC family structures. Altogether, these data indicate that CdpR NTR has a novel fold among AraC family, and its functions need to be further investigated.

### Unique CdpR HTH Motif and HTH-DNA Recognition

**Fig 6:**
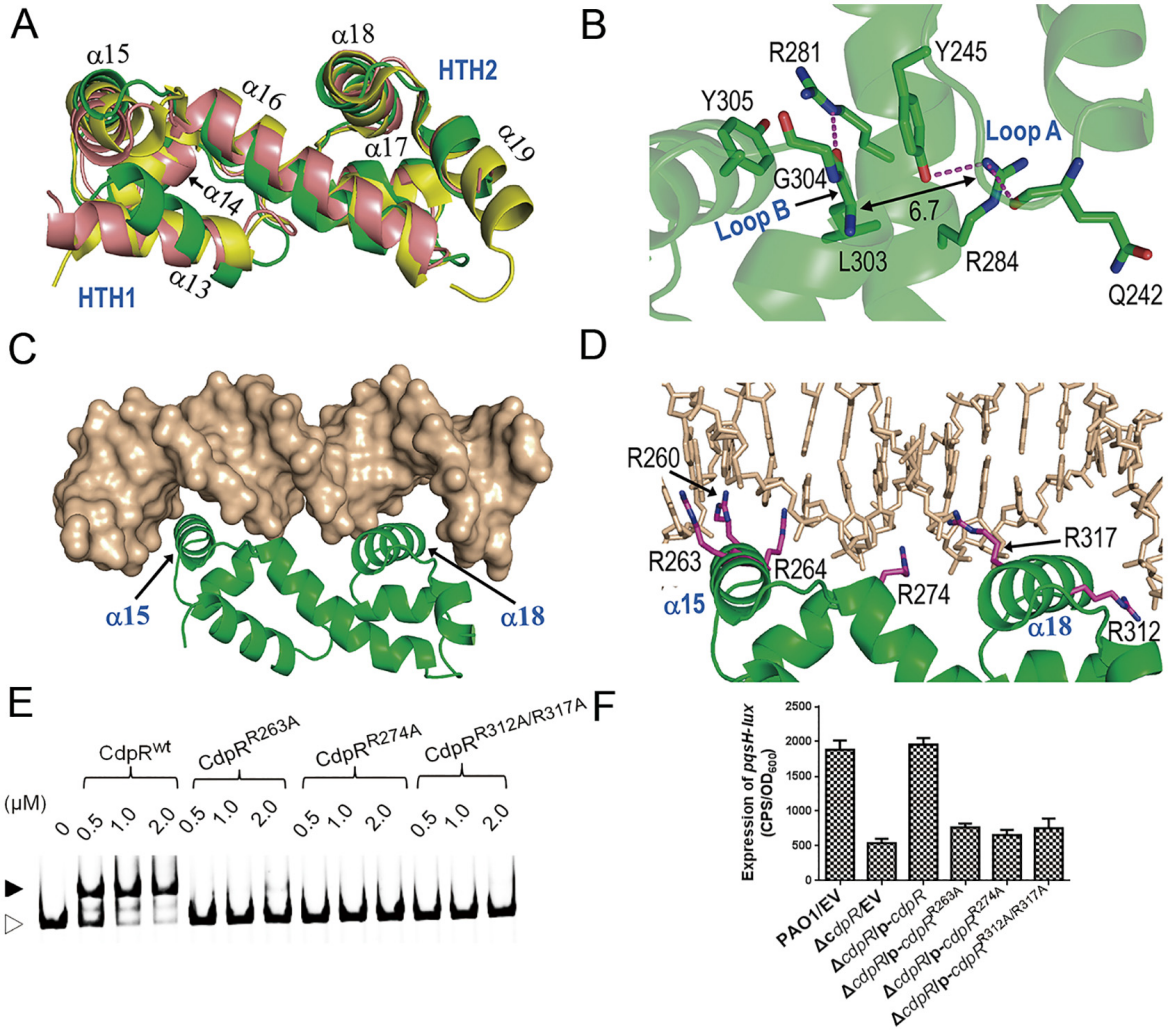
The overall structure of *Pa*CdpR HTH domain and *Pa*CdpR HTH-DNA model. **(A)** Structural comparison between *Pa*CdpR HTH, *Ec*MarA and sgAdpA, which are colored in green, purple, and yellow, respectively. α15 and α18 are the two DNA recognition helices of *Pa*CdpR. **(B)** The unique interactions between HTH1 and HTH2 in *Pa*CdpR. **(C** and **D)** The *Pa*CdpR HTH–DNA model showing the possible *Pa*CdpR HTH–DNA interaction. The DNAs and protein backbone were colored in beige and green, respectively. The side chains of protein residues, which may participate in DNA recognition, are shown as stick in atomic color (C: magenta; N: blue). **(E)** EMSAs showed that CdpR^R263A^, CdpR^R274A^, and CdpR^R312A/R317A^ could not bind to the *pqsH-cdpR* intergenic region. PCR products containing *pqsH*-cdpR intergenic region were added to the reaction mixtures at a concentration of 40 nM. The protein concentration (μM) for each sample is indicated above its lane. Open arrowhead, unbound DNA, filled arrowhead, CdpR-DNA complexes. **(F)** The expression of *pqsH-lux* was evaluated in the wild-type PAO1, the *cdpR* mutant and the Δ*cdpR* strain containing p-*cdpR*, p-*cdpR* ^R263A^, p-*cdpR* ^R274A^, or p-*cdpR* ^R312A/317A^, respectively. Results represent the average of triplicate experiments. Error bars indicate SDs. EV represents empty vector.

HTH domains are conserved in AraC family. Besides CdpR, the HTH domain structures of *Ec*AraC (PDB ID: 2K9S) [24], *Ec*MarA (multiple antibiotic resistant regulon A, PDB ID: 1BL0) [25], and *Streptomyces griseus* AdpA (PDB ID: 3W6V) [26] have also been reported. Although the overall folds are similar, structural comparison revealed that the detailed conformation of CdpR HTH is different from other AraC proteins (Fig 6A). CdpR HTH contains seven α helices (α13–α19), which can be further divided into two subdomains (S5A Fig), i.e., HTH1 (aa 229–271) and HTH2 (aa 291–329). The largest difference occurs at the HTH1 motif, especially the helix α13. Consistently, the sequence similarity at this region is also very low (S5B Fig).

The interaction modes between HTH1 and HTH2 motifs are also different within these structures. In *Ec*MarA and *sg*AdpA structures, loop A and loop B interact with each other via backbone van der Waals contacts; however, as depicted in Fig 6B, such interaction was not observed in CdpR, indicated by the shortest distance (6.7 Å) between the loop backbones. Instead, CdpR HTH1 and HTH2 mainly interact with each other through the backbone of loop B (aa 303–304) and the side chain of Tyr245 from loop A. This interaction was further enhanced by Arg281 and Arg284 of helice α16, which connects HTH1 and HTH2 and Tyr305 of loop B, via H-bonding and stacking. Both Tyr245 and Arg284 of CdpR are not conserved in other AraC-family proteins.

HTH domains are responsible for target DNA recognition, and the complex structures have been solved for *Ec*MarA and *sg*AdpA. To better understand the mechanism of CdpR-DNA recognition, we also carried out molecular modeling study. CdpR HTH may recognize the DNA base pairs using two positive charged residues (Arg260 and Arg264), corresponding to Arg262 and Arg266 of *sg*AdpA. In *sg*AdpA HTH–DNA structure, DNA backbones form several H-bonds with the side chains of positive charged residues (Fig 6C and 6D). Arg312 and Arg317 of CdpR HTH are corresponding to Arg315 and Arg320 of *sg*AdpA. The other four residues (Arg261, Arg269, Arg309, and His317) of *sg*AdpA are not conserved, but Arg263 of CdpR may replace Arg261 of *sg*AdpA during DNA recognition, supported by the similar orientations of their guanine groups. Structural analysis did not reveal any residues of CdpR, which could form H-bonds with DNA similar to Arg269, Arg309, and His317 of *sg*AdpA; lack of extensive interactions with DNA backbones may be the main cause of the weak CdpR-DNA binding affinity (Fig 6D).

In order to experimentally confirm the proposed structural model for CdpR-DNA complex, conserved residues predicted to contact DNA (Arg263, Arg274, Arg312, and Arg317) were mutated to Ala. We next performed EMSAs for comparing the ability of purified wild-type CdpR to bind a DNA probe encompassing the *pqsH*-*cdpR* intergenic region with those of its mutated derivatives. As shown in Fig 6E, CdpR^wt^ was able to shift about 50% of the probe at a concentration of 0.5 μM, while CdpR^R263A^, CdpR^R274A^, and CdpR^R312A/R317A^ were unable to shift the same DNA probe even at a concentration of 2 μM. To future confirm these residues are important for CdpR activity, we tested the expression of *pqsH-lux* in the *cdpR* mutant containing the expressing plasmid p-*cdpR* ^R263A^, p-*cdpR* ^R274A^, or p-*cdpR* ^R312A/317A^. As shown in Fig 6F, mutation of these residues did not restore the *pqsH* activity of *cdpR* mutant to wild-type levels. These results clearly suggest that these residues are important for CdpR activity and confirm the CdpR-DNA model is accurate.

### Identification of Mutants with Altered CdpR Expression

In order to identify regulators of *cdpR*, its promoter was used as a reporter (CTX-*cdpR*-*lux*) and subjected to transposon mutagenesis. After three rounds of screening of 8,000 mutants, 4 positive and 10 negative mutants were selected. Genes inserted by transposon were determined via arbitrary primed PCR and subsequent DNA sequencing (S2 Table). Interestingly, mutation of *lasI* compromised the expression of *cdpR* compared to wild-type PAO1, suggesting that LasI is a positive regulator of *cdpR*. The expression of *cdpR* was increased in the M6 (*cdpR*::*Tn*) strain compared to the wild-type strain, which is consistent with the observation in the *cdpR* deletion mutant (S6A Fig). As expected, pyocyanin production was also elevated in the M6 strain (S6B Fig). We noted that *vqsM* was missing in our screen, which might result from limited number (8,000) of mutants.

Of these genes, we are most interested in the ATP-dependent protease ClpP, which associates with ClpS (ATP-dependent Clp protease adaptor protein) and ClpA (ATP-binding protease component). These mutants showed very strong phenotypes, revealing that they are involved in CdpR-controlled pathway. Moreover, previous study has shown that ClpP is related to bacterial virulence [27]. *clpS* and *clpA* share the same operon [28]. To further verify the effects of the *clpS*/*clpP* transposon insertion, we made a *clpS-clpA* double mutant (Δ*clpS*Δ*clpA*) and a *clpP* single mutant (Δ*clpP*). As expected, the activity of *cdpR-lux* in these two deletion strains was higher than that in the wild-type strain (S7A Fig).

We next wanted to verify whether the protein level of CdpR was affected by these proteases. To this end, we constructed the CTX-*cdpR-flag* fusion and the activity of CdpR-*flag* was shown the same as the wild-type CdpR (S7B Fig). Next, we tested the expression of CdpR-Flag in the wild-type PAO1, the Δ*clpS*Δ*clpA* mutant, the Δ*clpP* mutant, and their complemented strains (Δ*clpS*Δ*clpA/p-clpSclpA*, and Δ*clpP/p-clpP*) using western-blot assay. Higher levels of CdpR-Flag were detected in the Δ*clpS*Δ*clpA* or Δ*clpP* mutant than in the wild-type strain. The complemented strains restored CdpR-Flag to the wild-type levels (S7C Fig). As expected, the expression of *pqsH* in Δ*clpS*Δ*clpA* or Δ*clpP* mutants was also higher than the wild-type PAO1 (S8 Fig). Taken together, these results indicate that ClpS/ClpA and ClpP negatively regulate the expression of CdpR.

### CdpR Interacted with Adaptor ClpS and Was Degraded by ClpAP Protease

**Fig 7:**
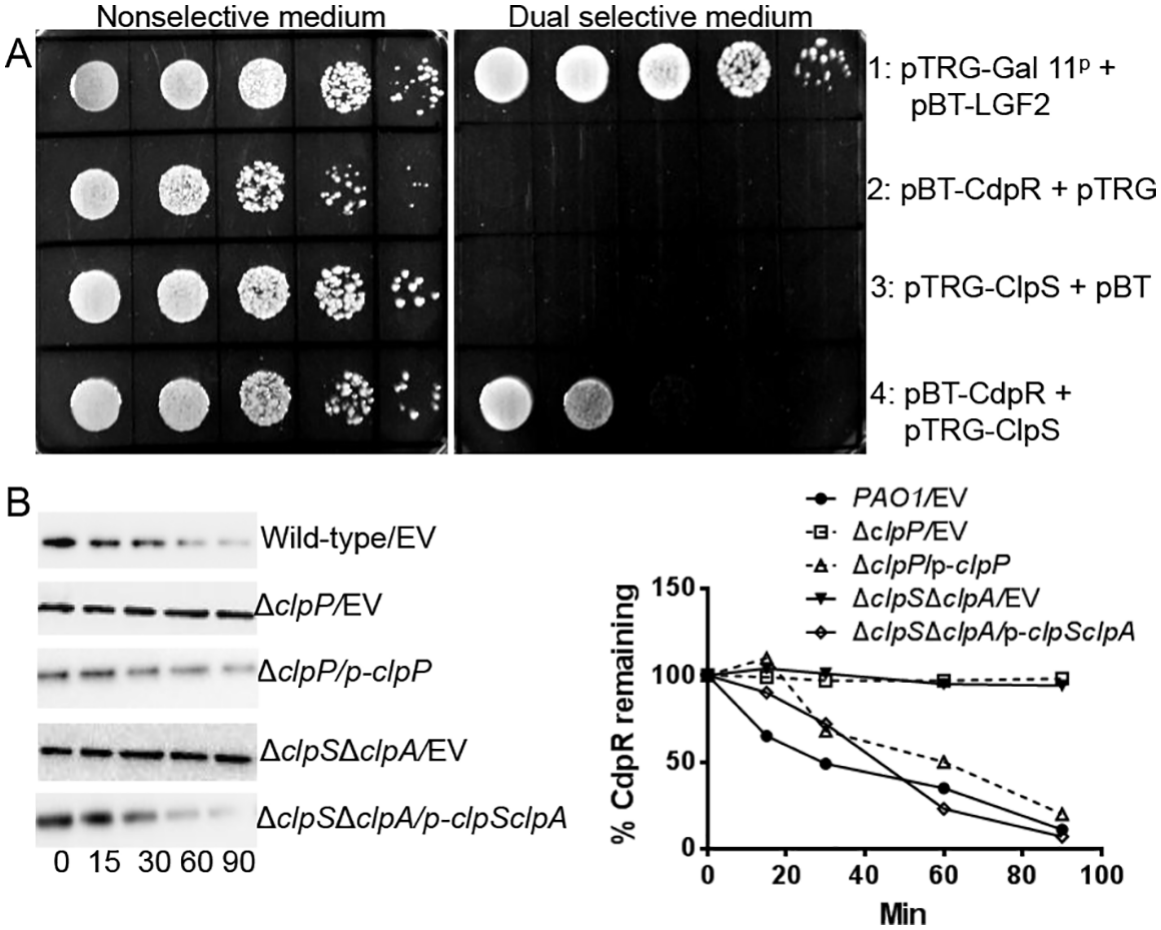
CdpR interacted with the adaptor protein ClpS and is degraded by ClpAP protease. **(A)** *E*. *coli* two-hybrid assay reveals an interaction between CdpR and ClpS. The recombinant strains harboring different proteins were separately streaked on nonselective and dual-selective media (3-amino-1, 2, 4-triazole + streptomycin). The strain expressing LGF2 and GAI11^P^ was used as a positive control. **(B)** Western-blot results of CdpR-flag protein levels in wild-type PAO1, Δ*clpP*, Δ*clpS*Δ*clpA*, and their complemented strains after translation blocking by chloramphenicol. Strains were grown to OD_600_ = 0.4 before treatment with chloramphenicol. Samples were taken as indicated time point. The experiments were repeated three times, with representative blots displayed. EV represents empty vector.

Given that ClpS is a bacterial adaptor that recognizes and delivers N-degron (N-terminal amino acids) substrates to the ClpAP or ClpCP AAA+ proteases [29,30], we hypothesized that ClpS interacts with CdpR by a bacterial two-hybrid system. Like strain 1 (the positive control), strain 4 (bearing pBT-CdpR and pTRG-ClpS) also grew on dual-selective medium (Fig 7A), indicating a direct interaction between CdpR and ClpS.

The direct interaction between ClpS and CdpR led us to examine if the CdpR protein can be degraded by the ATP-dependent ClpAP protease in vivo. We transformed mini-CTX-*cdpR*-flag into the wild-type PAO1, the Δ*clpP*, the Δ*clpS*Δ*clpA*, and their complemented strains. Western-blotting analyses of cell lysates were performed over the course of 90 min. Degradation of CdpR-Flag was observed in the wild-type PAO1 and the complemented strains with a half-life of less than 30 min (Fig 7B). However, CdpR levels remained steady in the Δ*clpP* and the Δ*clpS*Δ*clpA* strains (the second and fourth panels in Fig 7B), demonstrating that the ClpSA-ClpP protease system is responsible for CdpR degradation.

### ClpS and ClpAP Modulated *P*. *aeruginosa* Pathogenicity

**Fig 8:**
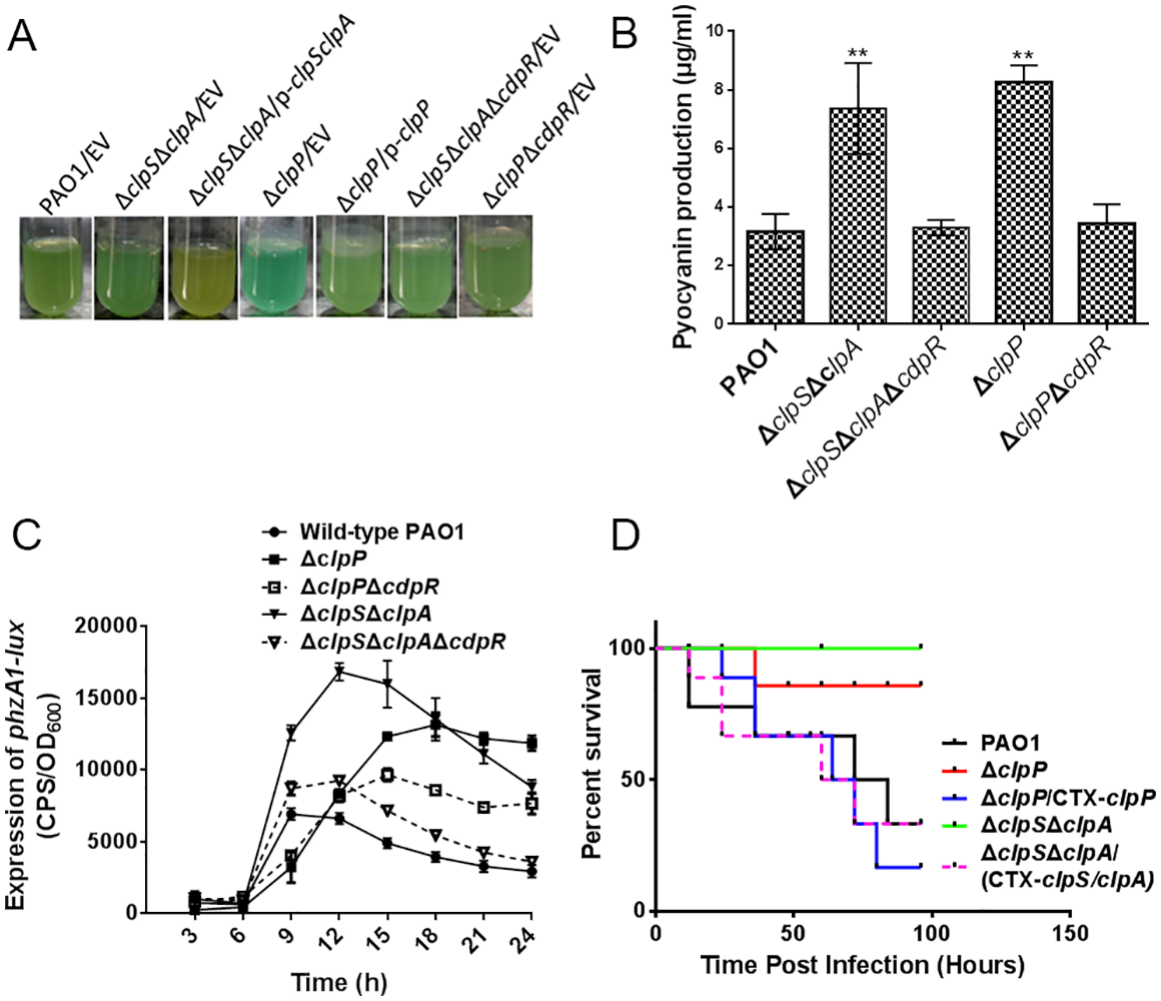
The effects of ClpP and ClpS/ClpA on *P*. *aeruginosa* virulence. **(A** and **B)** The production of pyocyanin was evaluated in wild-type PAO1, Δ*clpS*Δ*clpA*, Δ*clpP*, Δ*clpS*Δ*clpA*Δ*cdpR*, Δ*clpP*Δ*cdpR*, and their complemented strains. **p < 0.05 based on Student’s *t* test. EV represents empty vector. **(C)** The expression of *phzA1-lux* in wild-type PAO1, Δ*clpS*Δ*clpA*, Δ*clpP*, Δ*clpP*Δ*cdpR* and Δ*clpS*Δ*clpA*Δ*cdpR* strains. Results represent means ± SD, and data are representative of two independent experiments. **(D)** Deletion of *clpP* or *clpS/clpA* in *P*. *aeruginosa* decreased the pathogenicity in a mouse model. C57BL6 mice were intranasally challenged with wild-type PAO1, Δ*clpP*, Δ*clpS*Δ*clpA*, and the complemented strains (Δ*clpP/CTX-clpP*, Δ*clpS*Δ*clpA/CTX-clpSclpA*) at 1x10^7^ cfu in 50 μL PBS, and moribund mice were killed to obtain survival data (Kaplan-Meier Curve with Log-Rank test, *p* = 0.0043 (Δ*clpP*), *p* = 0.0028 (Δ*clpS*Δ*clpA*), *n* = 6).

A group of proteases has been shown to modulate *P*. *aeruginosa* virulence. For example, the deletion of Lon protease exhibits a defect in cell division and virulence-related properties, such as swarming, twitching, and biofilm formation [31]. It was also demonstrated that the *lon* mutant was less virulent in a mouse acute lung infection model as well as in a rat model of chronic infection [32]. The protease ClpP is also involved in antibiotic resistance and virulence-related phenotypes, such as motility and biofilm formation [17]. In the present study, ClpS/ClpA and ClpP controlled the expression of CdpR, which negatively regulated pyocyanin production and bacterial virulence (Fig 1). We showed that the Δ*clpS*Δ*clpA* and Δ*clpP* mutants produced more pigments than the wild-type PAO1 (Fig 8A and 8B). The deletion of *cdpR* in the Δ*clpS*Δ*clpA* or Δ*clpP* mutants restored pigment production and *phzA1-lux* activity to the wild-type level (Fig 8C). We also observed that the swarming motility of the Δ*clpA*Δ*clpS* and the Δ*clpP* strains could be partially reversed by CdpR (S9 Fig). Taken together, these results indicate that ClpSA-ClpP plays important roles in regulating pigment and motility. CdpR interacts with these intracellular proteases with important functions.

These altered virulence-associated phenotypes in the Δ*clpP* and Δ*clpS*Δ*clpA* mutants led us to evaluate the pathogenicity of these mutants in a mouse model. We found that deletion of *clpS/clpA* or *clpP* improved mouse survival compared to the wild-type PAO1. The Δ*clpS*Δ*clpA* and Δ*clpP* strains exhibited 100% and 85% of mice survival after 96 h, whereas the wild-type bacteria caused 70% death at 80 h (Fig 8D). These results demonstrated that ClpS/ClpA and ClpP proteases play important roles in *P*. *aeruginosa* pathogenicity in a mouse model.

## Discussion

Here, we present the first characterization of CdpR as an important regulator of QS system that directly binds to the promoter region of *pqsH*. We also solved the crystal structure of CdpR and revealed that CdpR NTD has a unique fold that is different from other AraC-family proteins. In addition, we found that CdpR interacts with ClpS before degradation by ClpAP proteases, thus regulates the *P*. *aeruginosa* virulence.

Although the QS systems in *P*. *aeruginosa* have been widely characterized in the last several decades, the complete signaling network and interactions with other systems still remain elusive. Previously, we showed that the QS regulator VqsM interacts with the promoter of *cdpR* [18]. Like VqsM, CdpR belongs to the AraC-family transcriptional regulators, which control a variety of cellular processes in bacteria, including carbon metabolism, stress responses, and virulence [21]. We performed a ChIP-seq assay that revealed 28 CdpR targets in the *P*. *aeruginosa* genome. More importantly, CdpR directly regulates the PQS system by binding to the promoter of *pqsH* (Fig 3 and S3 Fig). The Δ*cdpR* mutant exhibited higher pyocyanin and biofilm production as well as reduced bacterial motility (Fig 1), which are controlled by the PQS system [9,33]. Moreover, CdpR plays important roles in regulating bacterial pathogenicity in a mouse experiment. Mice inoculated with the Δ*cdpR* mutant showed more bacterial loads than mice infected with the wild-type strain (Fig 4A). The increased pathogenicity corresponds to the higher production of pyocyanin in the Δ*cdpR* mutant (Fig 1A).

Like the other AraC-family proteins, CdpR has an NTD whose function is deemed as binding to small molecules and a HTH region, which binds to a special substrate. From our ChIP-seq results, CdpR can target many genes, but its DNA binding affinity is relatively lower than other identified AraC proteins. This is due to the lack of positively charged residues in HTH domain that can interact with the DNA backbones (Fig 6 and S5 Fig). Unlike other AraC-family proteins that can bind arabinose using NTD domain and alter the gene regulation activity, CdpR has a novel NTR region, and its interaction with target DNAs is not affected by arabinose (S4 Fig). CdpR NTR may also play a role in the gene regulation via the interaction with other proteins or small molecules. Besides, analysis of the CdpR structure revealed that the conformation of CdpR HTH is different from other AraC proteins (Fig 6A). The proposed structural model for CdpR-DNA complex demonstrated that Arg263, Arg274, Arg312, and Arg317 are important for DNA-binding ability of CdpR (Fig 6D), which has been verified by in vitro EMSA and in vivo complemented assays where these residues were mutated (Fig 6E and 6F). Overall, our results provide insights into the function of AraC-family protein in different bacteria.

To further understand the regulatory mechanism upstream of CdpR, we performed a transposon mutagenesis assay that revealed several mutants showing altered expression of *cdpR* (S2 Table). The expression of *cdpR* was highly compromised in a mutant containing a transposon insertion in *clpAP*, which encodes ATP-dependent proteases [34]. Proteases ClpAP and Lon are involved in bacterial virulence expression, antibiotic resistance, and metabolism [27]. Lon protease negatively modulates QS by degrading autoinducer synthase LasI [35]. Our present study showed that CdpR interacted with the adaptor ClpS, and CdpR protein was degraded by the ClpAP protease in vivo (Fig 7). To the best of our knowledge, this is the first evidence showing the substrates of ClpS-ClpAP in *P*. *aeruginosa*. Further work needs to focus on the precise binding sites between ClpS and CdpR.

**Fig 9:**
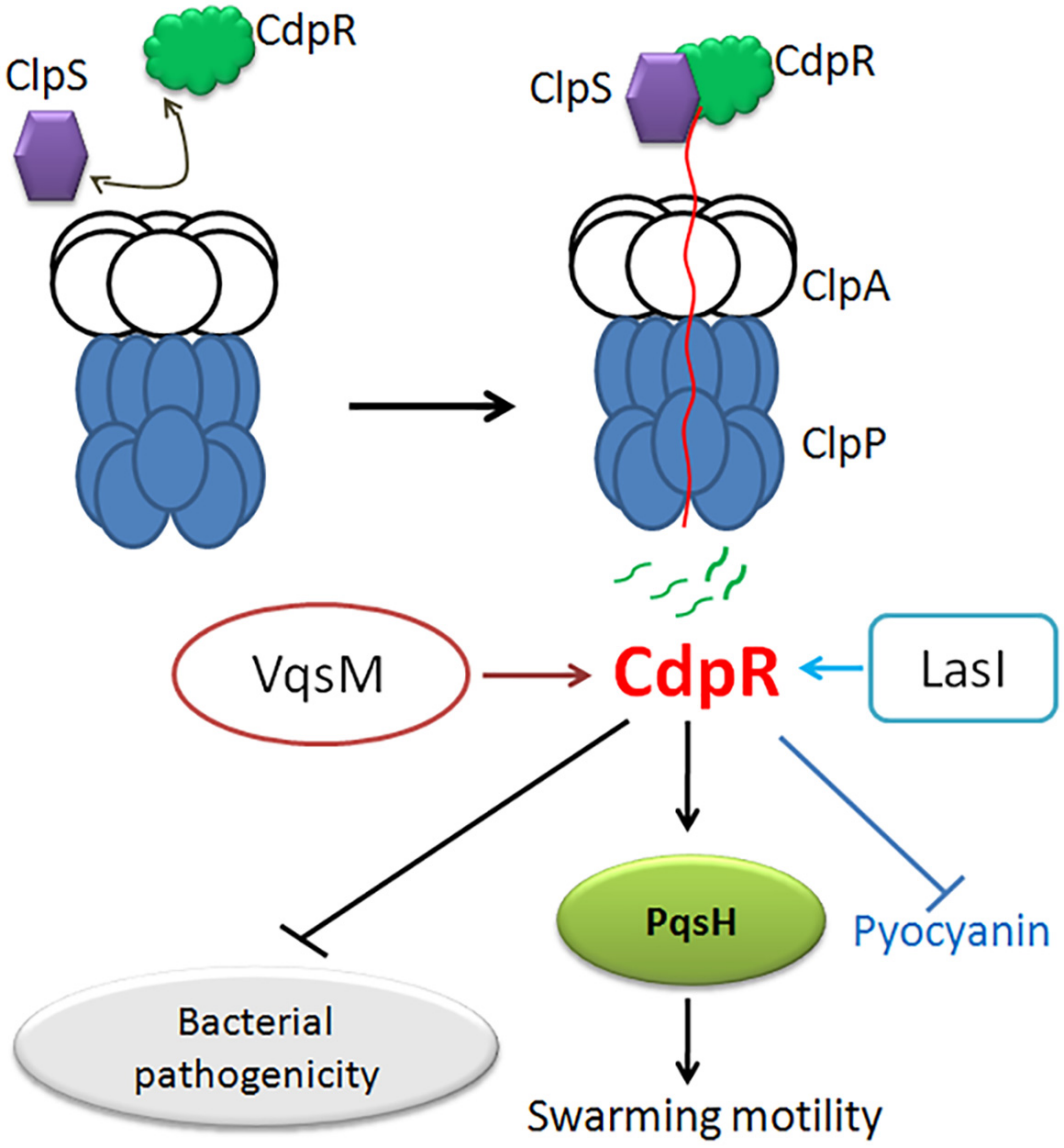
Schematic diagram of CdpR’s involvement in virulence expression and degradation by ClpAP protease in *P*. *aeruginosa*. The regulatory pathways of CdpR are proposed based on our current observations and previous studies. The PQS system positively regulates pyocyanin production and bacterial pathogenicity. In the present study, we demonstrated that CdpR directly bound to the *pqsH* promoter region and regulated the expression of *pqsH*. The adaptor ClpS interacted with CdpR protein, and associated with ClpAP to selectively degrade CdpR. Degradation of CdpR caused increased pyocyanin production, elevated the degree of biofilm formation and enhanced bacterial virulence in a mouse model. Solid arrows indicate positive regulation and solid T-bars present negative regulation.

In conclusion, we identified an AraC-family regulator, CdpR, which negatively modulates bacterial virulence (Fig 9). CdpR is a substrate of ATP-dependent ClpAP protease. CdpR also regulates the expression of *pqsH* by directly binding to its promoter regions. CdpR is positively modulated by LasI (S2 Table) and VqsM [18]. CdpR may work with other negative QS regulators, such as QscR and RsaL, to negatively regulate QS. In addition, ClpS interacts with CdpR and then associates with ClpAP to degrade CdpR. CdpR might indirectly regulate the expression of virulence factors, thus negatively controls *P*. *aeruginosa* pathogenicity. This work provides insight into roles of proteases in QS and bacterial virulence, which provides more cues to design effective antimicrobial drugs for the prevention of *P*. *aeruginosa* infections in the future.

## Materials and Methods

### Ethics Statement

All the experiments involving animals were approved by the University of North Dakota Institutional Animal Care and Use Committee and performed in accordance with the animal care and institutional guidelines (IACUC approval #: 1204–5) (Assurance Number: A3917-01). Animal experimental procedures, including treatment, care, and end point choice, followed Animal Research: Reporting in Vivo Experiment guidelines. When moribund animals manifested distress and/or mortal signs, such as lethargy, weight loss (15%–25%), lack of eating or drinking, tissue rupture, or edema, we considered these as endpoints and humanely euthanized them immediately by CO_2_. Recovery of the animals was achieved without complications, but we monitored hourly until they were awake and active after procedures and then every 6 h. Animals were monitored daily throughout the experimental process. Once sedation was diminished, the mice acted normally and appeared playful. There generally was no apparent pain from the intranasal instilling procedure. If any pain sign occurred, a 1% solution of lidocaine was applied to lessen pain.

### Bacterial Strains and Culture Conditions

The bacterial strains and plasmids used in this study are listed in S3 Table. *P*. *aeruginosa* PAO1 and derivatives were grown at 37°C on LB agar dishes or in broth with shaking at 220 rpm. Antibiotics were used at the following concentrations: for *E*. *coli*, gentamicin (Gm) at 15 μg/ml, ampicillin at 100 μg/ml, and tetracycline 10 μg/ml; for *P*. *aeruginosa*, gentamicin (Gm) at 50 μg/ml in LB or 150 μg/ml in PIA (*Pseudomonas* Isolate Agar); Tetracycline at 150 μg/ml in LB or 300 μg/ml in PIA and Carbenicillin at 500 μg/ml in LB.

### Chromatin Immunoprecipitation-Sequencing (ChIP-seq)

The procedures of chromatin immunoprecipitation (ChIP) were modified from previously described studies [18,36]. Wild-type PAO1 strain carrying pAK1900 or pAK1900-CdpR-VSV was cultured in LB medium until OD = 0.6 and then crosslinked with 1% formaldehyde (final concentration) for 10 min at 37°C with shaking. 125 mM glycine (final concentration) was added into the culture to stop the crosslinking. Bacteria were centrifuged and washed three times with a Tris buffer (150 mM NaCl, 20 mM Tris-HCl, pH 7.5). The pellets were resuspended in 500 μl IP buffer (150 mM NaCl, 50 mM HEPES-KOH pH 7.5, 1 mM EDTA, 0.1% SDS, 1% Triton X-100, 0.1% sodium deoxycholate and mini-protease inhibitor cocktail (Roche), and the DNA was sonicated to 100–300 bp. The chromatins were centrifuged (12,000 rpm, 4°C), and the supernatant was saved. All samples were treated by protein A beads (General Electric) before adding 50 μl agarose-conjugated anti-VSV antibodies. Washing, crosslink reversal, and purification of the ChIP DNA were performed by following previous procedures [36]. We used agarose gel to cut DNA fragments between 150 and 250 bp, which were then used for library construction with NEXTflex ChIP-Seq Kit (Bioo Scientific). After sequencing the libraries on HiSeq 2000 system (Illumina), the reads were mapped to the genome of *P*. *aeruginosa* PAO1 with TopHat (Version 2.0.0) [37]. MACS software (version 2.0.0) were then used to call peaks [20], which was subjected to MEME analyses to calculate the specific CdpR-binding motif [38].

### Construction of Plasmid

Plasmids *p-cdpR*, *p-clpS*/*clpA*, and *p-clpP* were constructed respectively by amplifying fragments with the corresponding primer pairs (S4 Table) pAK-*cdpR*-A/pAK-*cdpR*-S, pAK-*clpS*-A/pAK-*clpA*-S, and pAK-*clpP*-A/pAK-*clpP*-S by PCR. The PCR products were digested with the indicated enzymes and cloned into PAK1900 [39]. For construction of CTX-*clpP* and CTX-*clpA/clpS*, the sequence including its promoter and coding region was PCR-amplified with primers: CTX-*clpP*-F/CTX-*clpP*-R and CTX-*clpA/clpS*-F/CTX-*clpA/clpS*-R. These fragments were respectively ligated with Mini-CTX-*lux* [40].

The mini-*cdpR*-*flag*-A/mini-*cdpR*-*flag*-S primers were used to amplify the *cdpR* gene that intended to fuse with a C-terminal Flag-tag. The indicated enzyme-digested PCR products were cloned into the corresponding enzyme sites of Mini-CTX-lacZ to generate either Mini-CTX-*cdpR*-*flag*.

The plasmid pMS402 carrying a promoterless *luxCDABE* reporter gene cluster was used to construct promoter-*luxCDABE* reporter fusions of the *cdpR* as previously described [18]. The *cdpR* promoter region was amplified by PCR using the primers *cdpR-lux*-F (with *Xho*I site) and *cdpR-lux*-R (with *Bam*HI site) (S4 Table). The PCR products were cloned into the pMS402, yielding p-*cdpR*-*lux*. Besides the plasmid-based reporter system, an integration plasmid CTX6.1 originating from plasmid mini-CTX-*lux* was used to construct chromosomal fusion reporter. The pMS402 fragment containing the kanamycin-resistance marker, the MCS, and the promoter-*luxCDABE* reporter cassette was then isolated and ligated to CTX6.1, yielding CTX-*cdpR-lux*. The plasmid generated was first transferred into *E*. *coli* SM10-λ *pir*, and the *P*. *aeruginosa* reporter integration strain was obtained using biparental mating as previously reported [41]. All constructs were sequenced to verify that no mutations occurred for these constructs.

### Construction of *P*. *aeruginosa* Δ*cdpR*, Δ*clpS*Δ*clpA*, Δ*clpP*, Δ*cdpR*Δ*pqsH*, Δ*cdpR*Δ*clpS*Δ*clpA*, and Δ*cdpR*Δ*clpP* Mutants

For construction of gene knockout mutants, a SacB-based strategy was employed as described in previous and our studies [42]. To construct the *cdpR* null mutant (Δ*cdpR*), PCRs were performed to amplify sequences upstream (1,975 bp) and downstream (1,242 bp) of the intended deletion. The upstream fragment was amplified from PAO1 genomic DNA using primer pair, pEX-*cdpR*-up-S, and pEX-*cdpR*-up-A, while the downstream fragment was amplified with primer pair, pEX-*cdpR*-down-S, and pEX-*cdpR*-down-A (S4 Table). The two PCR products were digested and then cloned into *Bam*HI/*Hin*dIII-digested gene replacement vector pEX18Ap, yielding pEX18Ap-*cdpR*. A 0.9 kb gentamicin resistance cassette cut from pPS858 with *Xba*I was cloned into pEX18Ap-*cdpR*, yielding pEX18Ap-*cdpR*-Gm. The resultant plasmids were electroporated into PAO1 with selection for gentamicin resistance. Colonies showing both gentamicin resistance and loss of sucrose (5%) susceptibility were selected on LB agar plates containing 50 μg/ml of gentamicin and 5% sucrose, which typically indicates a double-crossover event and thus of gene replacement occurring. The pEX18Ap-*cdpR*-Tc was constructed by a similar strategy as described above. A 2.3 kb tetracycline resistance cassette was amplified from integration vector mini-CTX-lacZ with primer pair Tc-S/Tc-A (with *Xba*I site) (S4 Table) for replacing the *cdpR* gene in PAO1. The Δ*cdpR* mutant was further confirmed by PCR. The *pqsH*, *clpS*/*clpA*, and *clpP* mutant was generated by a similar strategy with deletion of *cdpR* gene in PAO1.

For generating *cdpR/pqsH*, *cdpR/clpSclpA*, and *cdpR/clpP* deletion strains (Δ*cdpR*Δ*pqsH*, Δ*cdpR*Δ*clpS*Δ*clpA*, Δ*cdpR*Δ*clpP*), the *cdpR* gene in Δ*pqsH*, Δ*clpS*Δ*clpA*, and Δ*clpP* mutant was deleted by a similar strategy with plasmid pEX18Ap-*cdpR*-Tc. These resultant mutants were verified by PCR.

### Expression and Purification of CdpR Protein

The plasmid encoding the His-Sumo-*Pa*CdpR was constructed, through PCR reaction and DNA ligation, and transferred into *E*. *coli* strain BL21 DE3. The recombinant strains were cultured in 1L LB medium supplemented with 50 ug/ml kanamycin at 37°C. Protein expression was induced at OD_600_ ≈ 0.6 by addition of isopropyl β-D-1-thiogalacto-pyranoside (IPTG) with a final concentration of 0.2 mM. The induced cultures were then grown at 18°C for additional 18 h. The cells were harvested by centrifugation and resuspended in lysis buffer (20 mM Mes pH 6.5, 200 mM (NH_4_)_2_SO_4_, 200 mM NaCl, 25 mM Imidazole pH8.0) and then were lysed twice under high pressure via JNBIO homogenizer. The homogenate was clarified by centrifugation, and the supernatant was loaded onto Ni-NTA column (GE healthcare). The protein was eluted by elution buffer (20 mM Mes pH 6.5, 300 mM (NH_4_)_2_SO_4_, 150 mM NaCl, 500 mM Imidazole pH 8.0) using a stage-wise gradient. The fractions containing the recombinant His-Sumo-*Pa*CdpR protein were pooled and dialyzed against Buffer S (20 mM Mes pH6.5, 200 mM (NH_4_)_2_SO_4_, 200 mM NaCl, 1‰ β-Me) for three hours at room temperature with Ulp1 protease added. The cleaved sample was not stable during dialysis and additional 100 mM (NH_4_)_2_SO_4_, 100 mM NaCl, and 2‰ β-Me were added to redissolve the protein precipitation. The sample was loaded onto Ni-NTA column again to remove the cleaved His-SUMO tag. Target *Pa*CdPR protein was contained in the flow-through, and its purity was analyzed by SDS-PAGE. Protein was concentrated via Amicon-Ultra centrifugal device from Millipore and stored at -80°C freezer until use. Selenomethionine-substituted *Pa*CdPR were expressed in M9 medium supplemented with 60 mg/L Se-Met (J&K), its purification procedure is similar as the full-length native protein. The purity was verified by SDS-PAGE gel (S1A Fig).

### Crystallization and Data Collection

Crystallization was performed at 16°C using the Gryphon robot system from Arts Robbin Instrument Company, which identified the initial crystallization condition for full-length *Pa*CdpR. The condition is composed of 1.0 M (NH_4_)_2_HPO_4_ and 0.1 M Acetate (pH4.5), which gives very tiny rod-shaped crystals. Relative larger crystals of Se-*Pa*CdpR (0.1×0.1× 0.2 mm) were obtained after several runs of optimization. The protein concentration is 3 mg/ml, and the well solution is similar as that of native proteins. 0.001 M TCEP was added to the crystallization condition as additive. Crystals of Se-*Pa*CdpR were cryoprotected by soaking in the mother liquid supplemented with 20% glycerol for 30 sec and then were flash-frozen by liquid nitrogen. The X-ray diffraction data was collected on beamline BL17U at Shanghai Synchrotron Radiation Facility (SSRF) at cryogenic temperature, maintained with Cryogenic system. One single crystal was used, and data processing was carried out with HKL2000 [43]. The data collection and processing statistics were summarized in S5 Table.

### Structure Determination, Refinement and *Pa*CdpR HTH-DNA Complex Modeling

The structure of Se-*Pa*CdpR was solved using the SAD method with the Autosol program [44] embedded in the Phenix suite, which built an initial model that covered about 75% of the residues. The refinement was done using the Refmac5 program of ccp4 [45]; and during refinement, 5% of the data was randomly selected and set aside for free R-factor cross validation calculations. The 2*F*_obs_-*F*_calc_ and *F*_obs_-*F*_calc_ electron density maps were regularly calculated and used as a guide for the building of the missing amino acids and solvent molecules using Coot [46]. The R_work_ and R_free_ of the final model are 18.8% and 23.4%, respectively. The rmsd of bond and angle is 0.005 Å and 0.963°, respectively. Other refinement parameters were also summarized in S5 Table. The structure factors and atomic coordinates can be found in the Protein Data Bank with the access code 5CHH.

The complex structures have been solved for two AraC family proteins, *Ec*MarA (PDB code: 1BL0) and *sg*AdpA (PDB code: 3W6V). The overall structure of *Pa*CdpR HTH is more similar to *sg*AdpA HTH than *Ec*MarA HTH, with rmsds of 1.7 Å and 2.1 Å, respectively. In addition, the target DNAs of *sg*AdpA and *Pa*CdpR are GC rich, whereas it is AT rich for *Ec*MarA targeting DNA; therefore, the *sg*AdpA HTH–DNA structure was used as reference during the model building. The *Pa*CdpR HTH–DNA complex was modeled in three steps. In the *sg*AdpA HTH–DNA complex structure, two symmetry-related DNA duplexes interact with the two DNA recognition helices (which are α15and α18 in *Pa*CdpR) from one protein molecule; and the two DNA duplexes are not broken, due to the lacking of 5'-phosphate groups. Therefore, the first step was the generation of 5'-phosphate groups and bridging it with the 3'-OH of its neighboring nucleotide, resulting continuous DNA duplex. Second, the modified DNA was docked on the *Pa*CdpR HTH structure, via the superimposing of the modified *sg*AdpA HTH–DNA structure with *Pa*CdpR HTH. The rmsd between the overall structure of *Pa*CdpR HTH and *sg*AdpA HTH is 1.7 Å, based on 111 pairs of Catoms. Finally, the side chains of some residues, including Arg260, Arg263, Arg264, Arg265, Arg274, and Arg317, were adjusted to avoid the clashes between protein and DNA. No further conformational change or energy minimization was involved in the modeling.

### EMSA

CdpR proteins were mixed with DNA probes (S4 Table) in 20 μl of the gel shift-loading buffer (20 mM HEPES, pH 8.0, 100 mM NaCl, 0.5 mM dithiothreitol, 10% Glycerol, and 3 μg/ml sheared salmon sperm DNA). After incubation at room temperature for 20 min, the samples were analyzed by 6% polyacrylamide gel electrophoresis in 0.5×TBE (Tris/Boric Acid/EDTA) buffer at 90 V for 90 min. The gels were stained by SYBR GOLD dye and subjected to screen on a phosphor screen (Tanon 5500).

### Dye Primer-Based DNase I Footprint Assay

The DNA footprint assay was performed by following previous procedures [18,47]. A 588-bp DNA containing the *pqsH* promoter region (–500 to +88) was amplified with primers *pqsH-g*f (with a 6-FAM modification at the 5’) and *pqsH-g*r (S4 Table). Forty nM of 6-FAM-labeled *pqsH* promoter probe was incubated with 2 μM of CdpR in gel-shift loading buffer. The protein-DNA mixtures were then partially digested with 0.05 units of DNase I (NEB) for 5 min at 25°C. The reaction was quenched by 0.25 M EDTA and purified with phenol-chloroform-isoamylalcohol (25:24:1) and then QIAquick PCR Purification kit (Qiagen). Control samples were done without CdpR protein. The genotype samples were run with the 3730 DNA Analyzer, and viewed with Peak Scanner (Applied Biosystems).

### Transposon Mutagenesis

The PAO1 containing the CTX-*cdpR-lux* reporter fusion was subjected to transposon mutagenesis using the mariner transposon vector pBT20 [48]. Briefly, the donor strain (*E*. *coli* SM10-λ*pir*) containing pBT20 and recipient PAO1-containing CTX-*cdpR-lux* were scraped from overnight plates and resuspended in 1 ml of M9 minimal medium. The bacterial suspensions were adjusted to an OD_600_ of 40 for the donor and an OD_600_ of 20 for the recipient. Next, 25 μl of each donor and recipient were mixed together and spotted on a dry LB agar plate and incubated at 37°C for overnight. Mating mixtures were scraped and resuspended in 1 ml of M9 minimal medium. Transposon-mutagenized bacteria were selected by plating on PIA plates containing gentamicin at 150 μg/ml. A transposon mutant library was constructed by picking 20,000 colonies grown on these selective plates. The mutants with altered expression of CTX-*cdpR-lux* were selected. The transposon insertion sites were determined by arbitrary primed PCR and subsequent sequencing of the PCR product [49].

### Bacterial Two-Hybrid Assays

Bacterial two-hybrid experiments were accomplished using the BacterioMatch II Two-Hybrid System Vector Kit (Agilent Technologies). The fragments of CdpR were cloned into the bait vector pBT in order to create a fusion protein with λ repressor protein (λcI). The DNA fragment of ClpS was inserted into the vector pTRG in frame with the α-subunit of RNA polymerase. The pBT-derived plasmids and pTRG-derived plasmid were then cotransformed into the validation reporter strain. Then, the resulting strains, grown at 37°C in SOC medium for 90 min, were harvested by centrifugation (5,000 g, 2 min) and washed twice with M9 ^+^ His-dropout broth. The cells were diluted to different gradients, spotted on the nonselective screening medium and selective screening medium, and finally incubated at 30°C for 2–4 d. The colonies grown on the selective screening medium were selected and streaked on the dual screening medium for further verification. The cotransformant containing pBT-LGF2 and pTRG-Gal 11^P^ plasmids was used as the positive control.

### Luminescence Screening Assays

Expression of *lux*-based reporters from cells grown in liquid culture was measured as counts per second (cps) of light production in a Synergy 2 (Biotek) as previously described [18]. Overnight cultures were diluted to OD_600_ = 0.2 with fresh LB medium, and then cultured for another 2 h. The cultures were added into a black 96-well plate with a transparent bottom. Sixty μl of sterilized mineral oil was added to culture. Promoter activities bacterial growth (OD = 595) were continuously measured every 30 min for 24 h in a Synergy 2 Plate Reader (BioTek).

### In Vivo Degradation Assays and Western Blot Analysis

The in vivo degradation assay was carried out to assess the stability of CdpR-Flag protein in *P*. *aeruginosa* as previously described [50,51]. Briefly, overnight LB cultures of tested strains were diluted 100-fold in fresh 20 ml LB medium in an Erlenmeyer flask with a flask volume-to-medium volume ratio of 5:1, and were aerated by shaking at 220 rpm. When OD_600_ value of the culture reached to approximately 0.4, chloramphenicol (a final concentration at 75 μg/ml) was added to the culture in order to block the translation, and samples for western blot analysis were removed at the indicated times. For the detection of CdpR-Flag, overnight LB cultures of the indicated strains were 1:100 diluted with LB medium recultured until A_600_ = 0.6. One hundred μl cultures were washed and resuspended in 15 μl PBS buffer.

The samples were solubilized in the SDS-PAGE loading buffer (50 mM Tris-HCl, pH 6.8; 2% SDS; 0.1% bromophenol blue; 1% mercaptoethanol; 10% glycerol) and then heated at 100°C for 15 min. SDS polyacrylamide gel electrophoresis was carried out according to the method of Laemmli [52] using a 10% slab gel with a 5% stacking gel and transferred onto PVDF (Bio-Rad) membranes. And then incubated with a mouse anti-Flag antibody (AOGMA, AGM12165) or anti-RNAP (Neoclone, WP003), followed by a sheep anti-mouse IgG antibody conjugated to horseradish peroxidase (HRP) (Code^#^: NA931, GE Healthcare), respectively. The relative abundance was determined by densitometric analysis using ImageQuant software.

### Biofilm Formation Assay

Biofilm formation was measured in a static system as previously described [53], with minor modifications. Visualization of biofilm formation was carried out in 15-mL borosilicate tubes. Briefly, cells from overnight cultures were inoculated at 1:100 dilutions into LB medium supplemented with appropriate antibiotics and grown at 30°C for 10 h. Biofilms were stained with 0.1% crystal violet (CV) and tubes were washed with water to remove unbound dye. Quantification of biofim formation was performed in 24-well polystyrene microtiter plates. LB and appropriate antibiotics was inoculated to a final OD_600nm_ of 0.01. The plates were incubated for 8 h or 14 h at 30°C. Crystal violet was added to each tube and stained for 15 min prior to removal by aspiration. Wells were rinsed three times by submerging the tubes in distilled water, and the remaining crystal violet was dissolved in 1 ml of 95% ethanol. A 1 ml portion of this solution was transferred to a new polystyrene tube, and the absorbance was measured at 600 nm.

### Measurement of Pyocyanin Production

Pyocyanin was extracted from culture supernatants and measured using previously reported methods [54]. Briefly, 3 ml chloroform was added to 5 ml culture supernatant. After extraction, the chloroform layer was transferred to a fresh tube and mixed with 1 ml 0.2 M HCI. After centrifugation, the top layer was removed and its A_520_ was measured. The amount of pyocyanin, in μg/ml, was calculated using the following formula: A_520_/A_600_ × 17.072 = μg of pyocyanin per ml.

### PQS Production and Bioassay of C_4_-HSL Activity

Quantification of PQS production as described previously [55]. Briefly, singles colonies were inoculated in 10 ml LB for 16 h at 37°C. Cultures were diluted 1:100 into fresh media and grown for 24 h as above. A 500 μl aliquot of each culture was mixed with 1 ml of acidified ethyl acetate, vortexed vigorously for 2 min, and then centrifuged for 10 min at 16,000 × g. The organic phase was transferred to a fresh tube and dried to completion. The solute was dissolved in 50 μl of a 1:1 mix of acidified ethyl acetate: acetonitrile for analysis.

The autoinducer of the *rhl* system, C_4_-HSL, was measured using an *rhlA* promoter-based *P*. *aeruginosa*, pDO100 (pKD-*rhlA*) as previously described [56]. Two microlitres of test bacterial culture (OD600 = 1.0) were inoculated onto the seeded bioassay plates, and the plates were incubated at 37°C for 18 h. The dark halo zone around bacterial colonies indicates AHL activity.

### Mice Experiment

Overnight culture of bacteria were 1:100 diluted with fresh LB medium and recultured until OD_600_ = 0.6. Six-week-old female C57BL6 mice were bought from the Harlan Laboratory (Indianapolis, IN). The animal procedures have been approved by the University of North Dakota institutional animal care and use committee (UND IACUC). Mice were anesthetized with 40 mg/kg ketamine plus 5 mg/kg diazepam. 1 × 10^7^ CFUs of *P*. *aeruginosa* were intranasally instilled into mice and survival rates were calculated for every bacterial strain.

### Nitroblue Tetrazolium (NBT) Assay

AM cells were cultured in 96-well plates at 37°C with 5% CO_2_ overnight. NBT dye (Sigma) was added to AM cells following the manufacturer’s procedures. The yellow-colored NBT changes to blue when oxidized by superoxide from AM cells [57]. The plate was kept at room temperature overnight for complete formation of dye product, which is monitored by a plate reader at 560 nm. Each experiment was conducted in three repeats.

### Histological Analysis

After BAL procedures and serum collection, lung tissues were fixed in 10% formalin using a routine histologic procedure. Ten μl of BAL and serum were applied on microscope slide. The PMN numbers were counted under a light microscope using a Hema staining kit (Thermofisher). Homogenizations of lung tissues were done using liquid nitrogen then dissolved in RIPA buffer and sonicated (10 s sonication with 10 s intervals, 3 times) for next analysis. Tissue damage was determined by H&E staining in formalin-fixed tissues [57].

## Supporting Information

S1 Data Contains data for Figs 1A–1C, 3D, 4A–4E, 6F, 7B, 8B–8D, S3C and S3D, S6A and S6B, S7A, and S8A. (XLSX)

S1 Fig CdpR binds to *sphR* and *cerN* promoter regions. **(A)** SDS-PAGE gel of CdpR protein after Ni-NTA column affinity chromatography purification. Lane 1, standard protein markers, Lane 2, purified CdpR protein. **(B)** CdpR binds to the *sphR-sphA* intergenic region. **(C)** CdpR binds to the *cerN* promoter region from ChIP-seq analysis. **(D, E, F)** EMSA shows that CdpR binds to the promoter region of *shpR* and *cerN*, respectively, but not to *sphR* and *cerN* mutated fragments (without the binding motif). The promoter fragment of *lasR* is a negative control. PCR products containing *sphR*, *sphR-p1*, *cerN*, *cerN-p1*, or *lasR* promoter regions were added to the reaction mixtures at a concentration of 40 nM. CdpR protein was added to reaction buffer in lanes with 1.0, 0.5, 0.25, 0.125 μM, respectively. No protein was added in Lane 1.
(TIF)

S2 Fig **(A)** CdpR binds to some selected target regions in vitro. The chosen promoter regions (for *opdC*, *PA4772*, *PA4087*, *PA0440*, *PA3388*, *cysG*, *recC*, *pscC*, *PA5146*, and *PA1271valS*, *PA0159*, *PA4541*, *selB*, *serS*, *PA3992*, *PA5114*, and *PA4513*) and EMSA analyses are described in Materials and Methods. PCR products containing the indicated fragments were added to the reaction mixtures at approximately 40 nM each. The protein concentration (μM) for each sample is indicated above its lane. **(B)** Sequence of the *cerN* or *sphR* promoter region. The ATG starting codon is in boldface and highlighted by red. The predicted conserved sequence is underlined.
(TIF)

S3 Fig **(A)** Assessment of mutations or deletion of the protected region on CdpR binding. EMSA assays were performed using a wild-type fragment of the *pqsH* promoter (*pqsH-p*), a fragment with a deletion of the protected region (*pqsH-p1*), and a wild-type fragment with CTGCGCCTGGATGAT mutated to TGACTTCTGGATGAT (*pqsH-M*). Promoter fragments were added to the reaction mixtures at a concentration of 40 nM. CdpR protein was added to reaction buffer in lanes (2–5) with 0.25, 0.5, 1.0, 2.0 μM, respectively. No protein was added in Lane 1. **(B)** The C_4_-HSL production in the indicated strains. A C_4_-HSL plate bioassay was carried out using *P*. *aeruginosa* (PDO100*-rhlA*). Two μL bacterial test culture (OD_600_ = 1.0) were inoculated, and the plates were incubated at 37°C for 24 h. The halo zone around bacterial colonies indicates C_4_-HSL activity. The standard sample C_4_-HSL was used as the positive control. **(C)** Effects of mutations to the protected region on the promoter activity of *pqsH*. CTGCGCCTGGATGAT was mutated to TGACTTCTGGATGAT. Bacteria were grown in LB with 37°C and luminescence activity was evaluated. Results represent means ± SD, and data are representative of three independent experiments. **(D)** The expression of *cdpR-lux* was tested in the wild-type PAO1, the Δ*cdpR* mutant, and the Δ*cdpR* complemented strain. **p < 0.05 compared to wild-type or complemented strain by Student’s *t* test. EV represents empty vector.
(TIF)

S4 Fig The overall structure of *Ec*AraC-arabinose complex (A) and the impact of arabinose on the 23 bp-dsDNA binding by CdpR (B). (TIF)

S5 Fig The overall structure of *Pa*CdpR HTH **(A)** and the structure-based sequence alignment of *Pa*CdpR HTH with *Ec*AraC (PDB code: 2K9S), *Ec*MarA (multiple antibiotic resistant regulon A, PDB code: 1BL0), and *S*. *griseus* AdpA (PDB code: 3W6V). **(B)** The residues in gray are disordered in the structures.
(TIF)

S6 Fig CdpR is negatively autoregulated. **(A)** The expression of *cdpR-lux* was tested in wild-type PAO1, M6 (*tspR*::Tn), M6 (*tspR*::Tn) complemented strain M6, a Δ*cdpR* strain, and a Δ*cdpR* complemented strain. **(B)** The pyocyanin production was measured in the indicated strains. Images represent the pigment of the indicated strains after 24 h of growth with shaking in LB. **p < 0.05 compared to wild-type or complemented strain by Student’s *t* test. Results represent means ± SD, and data are representative of three independent experiments. EV represents empty vector.
(TIF)

S7 Fig The ClpS/A-ClpP proteases are required for *cdpR* activity. **(A)** The expression of *cdpR* was measured in the wild-type PAO1, the Δ*clpS*Δ*clpA* mutant, the Δ*clpP* mutant, and their respective complemented strains (Δ*clpS*Δ*clpA*/p-*clpSclpA*, Δ*clpP*/p-*clpP*). Results represent means ± SD, and data are representative of three independent experiments. **(B)** The swarming motility of the indicated strains. **(C)** Western blotting confirms that the expression of *cdpR* was drastically higher in Δ*clpS*Δ*clpA* and Δ*clpP* strains than in wild-type PAO1. The indicated strains containing the integrated single-copy plasmid CTX-*cdpR*-flag were cultured at OD_600_ = 0.6. The whole-cell extracts from the designated strains were subjected to SDS/PAGE separation and subsequent immuno-blotting. EV represents empty vector.
(TIF)

S8 Fig The ClpS/A-ClpP proteases control *pqsH* activity. **(A)** The expression of *pqsH* was measured in wild-type PAO1, Δ*clpS*Δ*clpA* mutants, Δ*clpP* mutant, and their complemented strains (Δ*clpS*Δ*clpA* /p-*clpSclpA*, Δ*clpP*/p-*clpP*), respectively. Results represent means ± SD, and data are representative of three independent experiments. **(B)** Western blotting shows that more *pqsH* was detected in Δ*clpS*Δ*clpA* or Δ*clpP* strains than in wild-type PAO1. The indicated strains containing the integrated single-copy plasmid CTX-*pqsH*-flag were cultured at OD_600_ = 0.6. The whole-cell extracts from the designated strains were subjected to SDS/PAGE separation and subsequent immunoblotting. EV represents empty vector.
(TIF)

S9 Fig The swarming motility of *clpS/clpA* or *clpP* mutant is partially dependent on CdpR expression. Overnight cultures of indicated strains were spotted onto swarming plates as 2 μL aliquots. After inoculation, the plates were incubated at 37°C, and images were captured after 14 h of growth. The experiments were repeated at least three times, and similar results were observed. EV represents empty vector.
(TIF)

S1 Table Whole-genome location analysis of CdpR from ChIP-seq. (DOCX)

S2 Table Mutated genes identified that altered the promoter activity of *cdpR* gene. (DOCX)

S3 Table Bacterial strains and plasmids used in this study. (DOC)

S4 Table Primers used in this study. (DOCX)

S5 Table Data collection and structural refinement statistics. (DOCX)

## Abbreviations

AHL: N-acyl-homoserine lactone
AM: alveolar macrophages
AQ: 2-alkyl-4-quinolone
BAL: bronchoalveolar lavage
CFU: colony-forming units
ChIP: chromatin immunoprecipitation
cps: counts per second
CR: connector region
*Ec*AraC: *E*. *coli* AraC
EMSA: electrophoretic mobility shift assay
HHQ: 2-heptyo-4(1H)-quinolone
HRP: horseradish peroxidase
HTH: helix-turn-helix
IPTG: isopropyl β-D-1-thiogalacto-pyranoside
LB: Luria-Beertani
MEME: multiple EM for motif elicitation
NBT: Nitroblue Tetrazolium
NTD: N-terminal domain
NTR: N-terminal region
PBS: phosphate buffered saline
PMN: polymorphonuclear neutrophil
PQS: *Pseudomonas* quinolone signal
QS: quorum-sensing
SD: standard deviation
SSRF: Shanghai Synchrotron Radiation Facility
