# Supplementary figures and images for: Structural and Molecular Mechanism of CdpR Involved in Quorum-Sensing and Bacterial Virulence in Pseudomonas aeruginosa
Source: PLoS Biol. 2016 Apr 27;14(4):e1002449. doi: 10.1371/journal.pbio.1002449 (PMC4847859; doi:10.1371/journal.pbio.1002449)

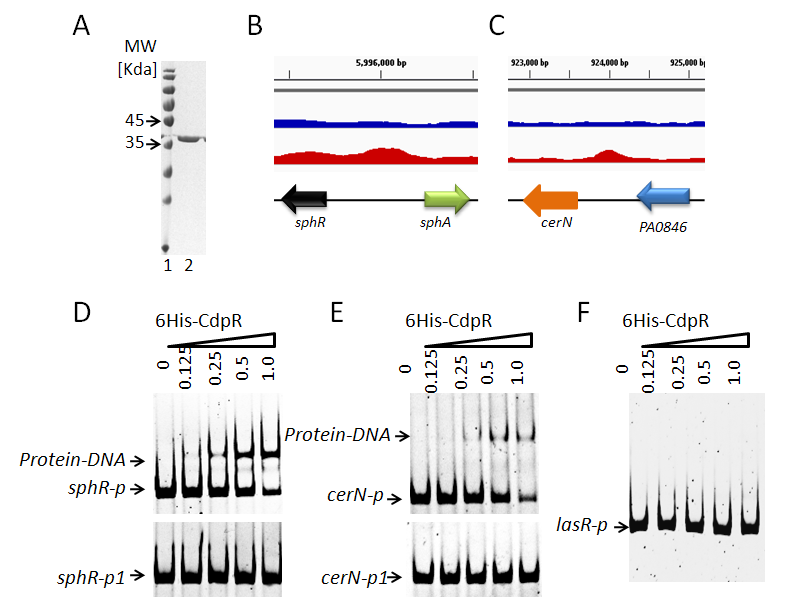

Supplement: S1 Fig — (A) SDS-PAGE gel of CdpR protein after Ni-NTA column affinity chromatography purification. Lane 1, standard protein markers, Lane 2, purified CdpR protein. (B) CdpR binds to the sphR-sphA intergenic region. (C) CdpR binds to the cerN promoter region from ChIP-seq analysis. (D, E, F) EMSA shows that CdpR binds to the promoter region of shpR and cerN, respectively, but not to sphR and cerN mutated fragments (without the binding motif). The promoter fragment of lasR is a negative control. PCR products containing sphR, sphR-p1, cerN, cerN-p1, or lasR promoter regions were added to the reaction mixtures at a concentration of 40 nM. CdpR protein was added to reaction buffer in lanes with 1.0, 0.5, 0.25, 0.125 μM, respectively. No protein was added in Lane 1. (TIF) [file pbio.1002449.s002.tif]

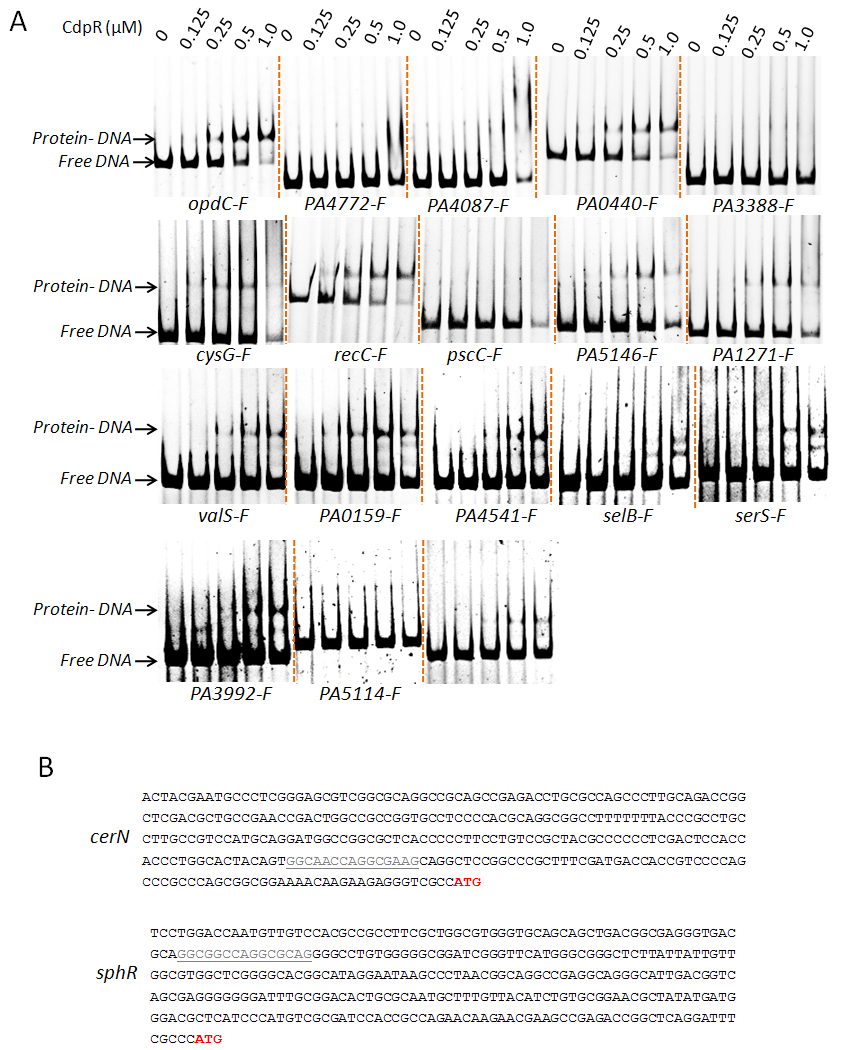

Supplement: S2 Fig — (A) CdpR binds to some selected target regions in vitro. The chosen promoter regions (for opdC, PA4772, PA4087, PA0440, PA3388, cysG, recC, pscC, PA5146, and PA1271valS, PA0159, PA4541, selB, serS, PA3992, PA5114, and PA4513) and EMSA analyses are described in Materials and Methods. PCR products containing the indicated fragments were added to the reaction mixtures at approximately 40 nM each. The protein concentration (μM) for each sample is indicated above its lane. (B) Sequence of the cerN or sphR promoter region. The ATG starting codon is in boldface and highlighted by red. The predicted conserved sequence is underlined. (TIF) [file pbio.1002449.s003.tif]

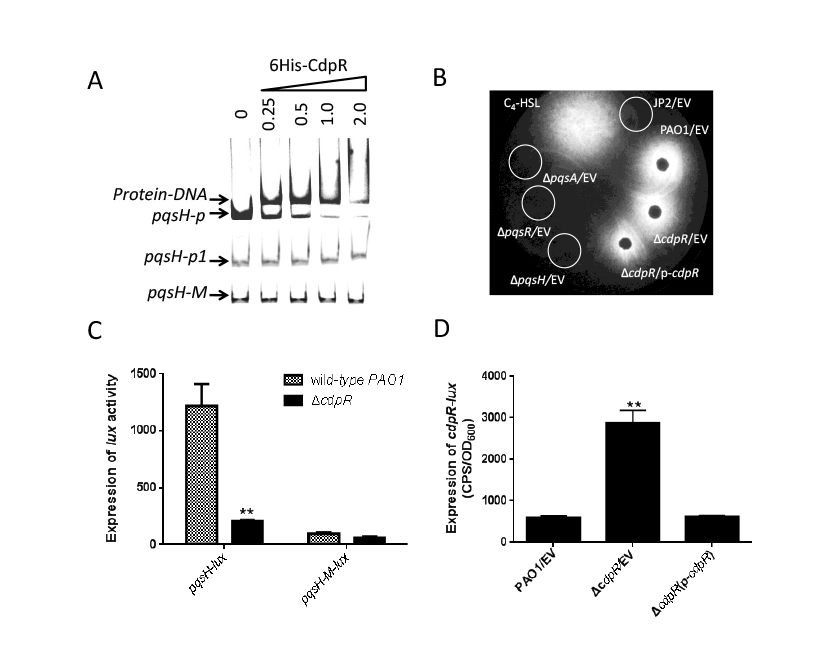

Supplement: S3 Fig — (A) Assessment of mutations or deletion of the protected region on CdpR binding. EMSA assays were performed using a wild-type fragment of the pqsH promoter (pqsH-p), a fragment with a deletion of the protected region (pqsH-p1), and a wild-type fragment with CTGCGCCTGGATGAT mutated to TGACTTCTGGATGAT (pqsH-M). Promoter fragments were added to the reaction mixtures at a concentration of 40 nM. CdpR protein was added to reaction buffer in lanes (2–5) with 0.25, 0.5, 1.0, 2.0 μM, respectively. No protein was added in Lane 1. (B) The C4-HSL production in the indicated strains. A C4-HSL plate bioassay was carried out using P. aeruginosa (PDO100-rhlA). Two μL bacterial test culture (OD600 = 1.0) were inoculated, and the plates were incubated at 37°C for 24 h. The halo zone around bacterial colonies indicates C4-HSL activity. The standard sample C4-HSL was used as the positive control. (C) Effects of mutations to the protected region on the promoter activity of pqsH. CTGCGCCTGGATGAT was mutated to TGACTTCTGGATGAT. Bacteria were grown in LB with 37°C and luminescence activity was evaluated. Results represent means ± SD, and data are representative of three independent experiments. (D) The expression of cdpR-lux was tested in the wild-type PAO1, the ΔcdpR mutant, and the ΔcdpR complemented strain. **p < 0.05 compared to wild-type or complemented strain by Student’s t test. EV represents empty vector. (TIF) [file pbio.1002449.s004.tif]

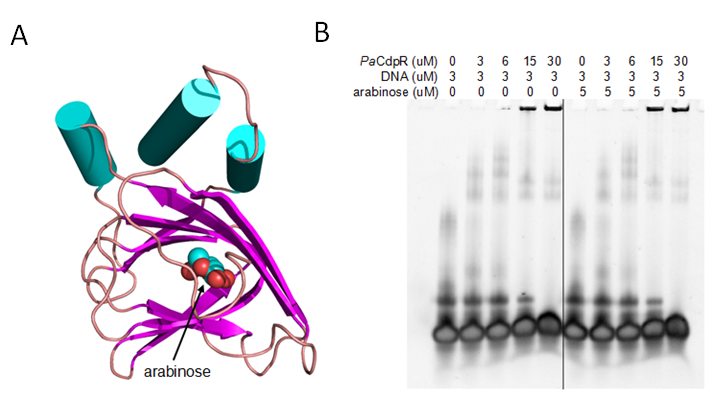

Supplement: S4 Fig — (TIF) [file pbio.1002449.s005.tif]

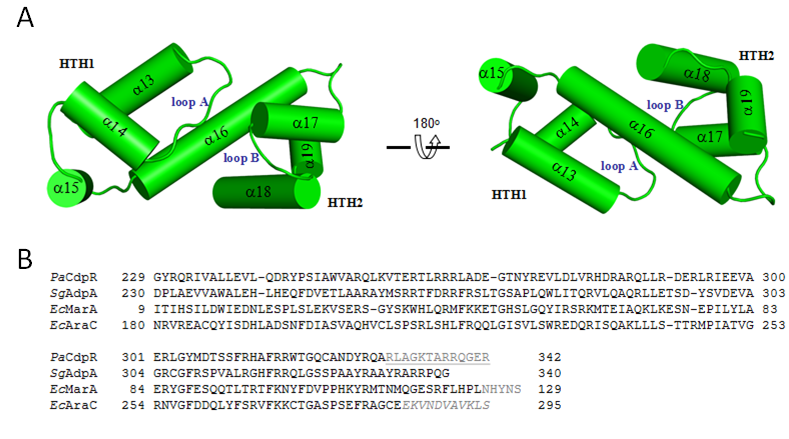

Supplement: S5 Fig — The overall structure of PaCdpR HTH (A) and the structure-based sequence alignment of PaCdpR HTH with EcAraC (PDB code: 2K9S), EcMarA (multiple antibiotic resistant regulon A, PDB code: 1BL0), and S. griseus AdpA (PDB code: 3W6V). (B) The residues in gray are disordered in the structures. (TIF) [file pbio.1002449.s006.tif]

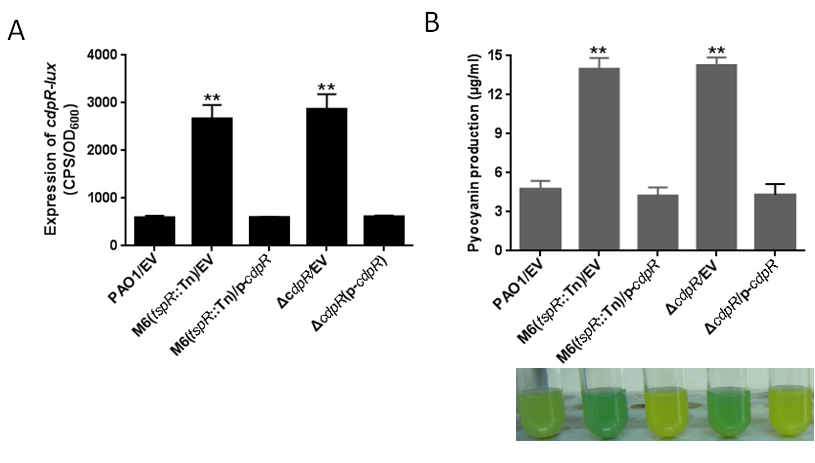

Supplement: S6 Fig — (A) The expression of cdpR-lux was tested in wild-type PAO1, M6 (tspR::Tn), M6 (tspR::Tn) complemented strain M6, a ΔcdpR strain, and a ΔcdpR complemented strain. (B) The pyocyanin production was measured in the indicated strains. Images represent the pigment of the indicated strains after 24 h of growth with shaking in LB. **p < 0.05 compared to wild-type or complemented strain by Student’s t test. Results represent means ± SD, and data are representative of three independent experiments. EV represents empty vector. (TIF) [file pbio.1002449.s007.tif]

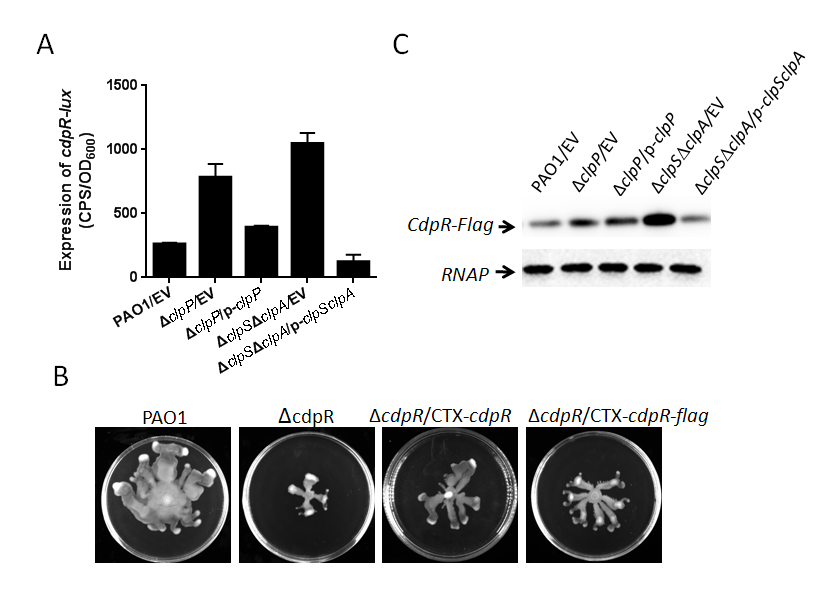

Supplement: S7 Fig — (A) The expression of cdpR was measured in the wild-type PAO1, the ΔclpSΔclpA mutant, the ΔclpP mutant, and their respective complemented strains (ΔclpSΔclpA/p-clpSclpA, ΔclpP/p-clpP). Results represent means ± SD, and data are representative of three independent experiments. (B) The swarming motility of the indicated strains. (C) Western blotting confirms that the expression of cdpR was drastically higher in ΔclpSΔclpA and ΔclpP strains than in wild-type PAO1. The indicated strains containing the integrated single-copy plasmid CTX-cdpR-flag were cultured at OD600 = 0.6. The whole-cell extracts from the designated strains were subjected to SDS/PAGE separation and subsequent immuno-blotting. EV represents empty vector. (TIF) [file pbio.1002449.s008.tif]

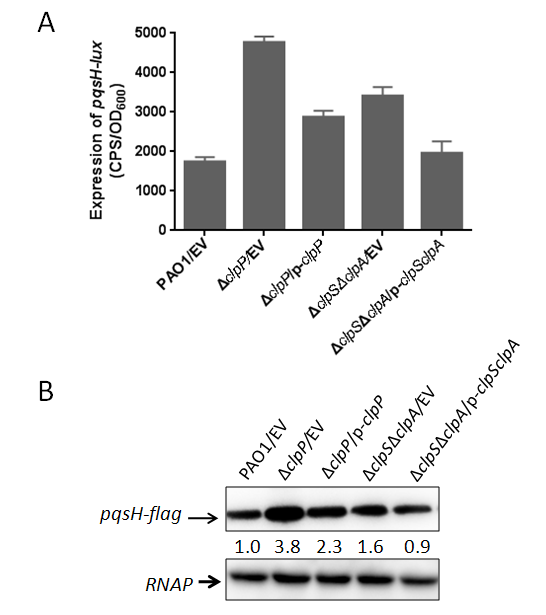

Supplement: S8 Fig — (A) The expression of pqsH was measured in wild-type PAO1, ΔclpSΔclpA mutants, ΔclpP mutant, and their complemented strains (ΔclpSΔclpA /p-clpSclpA, ΔclpP/p-clpP), respectively. Results represent means ± SD, and data are representative of three independent experiments. (B) Western blotting shows that more pqsH was detected in ΔclpSΔclpA or ΔclpP strains than in wild-type PAO1. The indicated strains containing the integrated single-copy plasmid CTX-pqsH-flag were cultured at OD600 = 0.6. The whole-cell extracts from the designated strains were subjected to SDS/PAGE separation and subsequent immunoblotting. EV represents empty vector. (TIF) [file pbio.1002449.s009.tif]

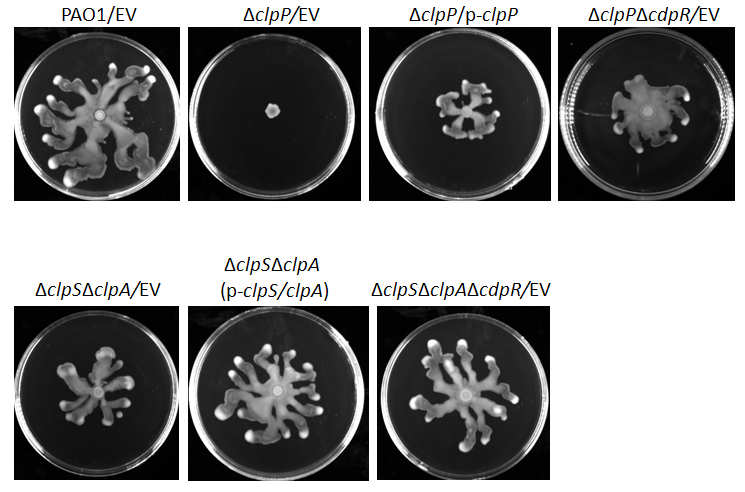

Supplement: S9 Fig — Overnight cultures of indicated strains were spotted onto swarming plates as 2 μL aliquots. After inoculation, the plates were incubated at 37°C, and images were captured after 14 h of growth. The experiments were repeated at least three times, and similar results were observed. EV represents empty vector. (TIF) [file pbio.1002449.s010.tif]
