# Supplementary material for: Structural and Molecular Mechanism of CdpR Involved in Quorum-Sensing and Bacterial Virulence in Pseudomonas aeruginosa
Source: PLoS Biol. 2016 Apr 27;14(4):e1002449. doi: 10.1371/journal.pbio.1002449 (PMC4847859; doi:10.1371/journal.pbio.1002449)
Supplement: S1 Table — (DOCX) [file pbio.1002449.s011.docx]

**Table S1**. Whole-genome location analysis of CdpR from ChIP-seq

| **start** | **end** | **length** | **abs_summit** | **fold_enrichment** | **gene** | **position** | **protein** | **category** |
| --- | --- | --- | --- | --- | --- | --- | --- | --- |
| 104759 | 104914 | 156 | 104838 | 1.79149 | *tagJ1* | overlap end | T4SS | secretion |
| 183342 | 183555 | 214 | 183508 | 1.65095 | *PA0159* | inside | probable transcriptional regulator | regulatory |
| 185642 | 185833 | 192 | 185721 | 2.28607 | *opdC* | inside | membrane protein | transport |
| 494218 | 494530 | 313 | 494389 | 3.66105 | *PA0440* | overlap end | glutamate biosynthsis | metabolic |
| 923831 | 924132 | 302 | 924002 | 3.26273 | *cerN* | upstream | ceramidase | hypothetical |
| 1330020 | 1330179 | 160 | 1330110 | 1.93287 | *PA1227* | overlap end | unknown | hypothetical |
| 1382625 | 1382887 | 263 | 1382752 | 3.05901 | *PA1271* | inside | recepter protein | transport |
| 1445768 | 1445963 | 196 | 1445852 | 2.7128 | *PA1334* | overlap start | FMN oxidoreductase | hypothetical |
| 1861196 | 1861348 | 153 | 1861247 | 2.30588 | *pscC* | inside | T3SS protein | virulence |
| 2927669 | 2929617 | 1949 | 2928320 | 5.00172 | *cdpR (PA2588)* | upstream | probable transcriptional regulator | virulence |
| 2952997 | 2953241 | 245 | 2953130 | 3.44186 | *cysG* | inside | siroheme synthase | metabolic |
| 2954372 | 2954549 | 178 | 2954464 | 1.88489 | *serS* | inside | ser-tRNA | metabolic |
| 3188894 | 3189101 | 208 | 3189005 | 1.82977 | *PA2835* | inside | membrane protein | transport |
| 3793338 | 3793537 | 200 | 3793428 | 2.69934 | *PA3388* | inside | methyltransferase | hypothetical |
| 4293325 | 4293523 | 199 | 4293429 | 2.016 | *valS* | inside | val-tRNA synthase | metabolic |
| 4472334 | 4472527 | 194 | 4472443 | 2.08546 | *PA3992* | inside | transferase | hypothetical |
| 4570532 | 4570817 | 286 | 4570689 | 2.29599 | *PA4087* | inside | unknown | hypothetical |
| 4808408 | 4808609 | 202 | 4808515 | 2.41033 | *recC* | inside | exodeoxyrbinulease V | Nucleic acids |
| 5051871 | 5052044 | 174 | 5051920 | 1.72941 | *PA4513* | inside | sulfite reductase | metabolic |
| 5085492 | 5085754 | 263 | 5085644 | 1.9442 | *PA4541* | inside | membrane protein | secretion |
| 5197615 | 5197785 | 171 | 5197715 | 1.98212 | *PA4629* | overlap end | membrane protein | hypothetical |
| 5358828 | 5358981 | 154 | 5358887 | 1.81605 | *PA4772* | inside | glycolate oxidase | metabolic |
| 5387133 | 5387313 | 181 | 5387134 | 1.58295 | *PA4802* | inside | amidohydrolase | metabolic |
| 5392869 | 5393065 | 197 | 5392989 | 1.69952 | *selB* | inside | translation elongation factor | metabolic |
| 5759902 | 5760056 | 155 | 5759970 | 1.85806 | *PA5114* | inside | membrane protein | hypothetical |
| 5795706 | 5795866 | 161 | 5795774 | 2.31992 | *PA5146* | inside | unknown | hypothetical |
| 5953822 | 5954016 | 195 | 5953919 | 2.09889 | *PA5290* | inside | unknown | hypothetical |
| 5995963 | 5996118 | 156 | 5996013 | 1.86562 | *sphR* | upstream | sphingosine-responsive regulator | virulence |
