# Supplementary material for: Structural and Molecular Mechanism of CdpR Involved in Quorum-Sensing and Bacterial Virulence in Pseudomonas aeruginosa
Source: PLoS Biol. 2016 Apr 27;14(4):e1002449. doi: 10.1371/journal.pbio.1002449 (PMC4847859; doi:10.1371/journal.pbio.1002449)
Supplement: S2 Table — (DOCX) [file pbio.1002449.s012.docx]

| Strains | Gene name  or PA no. | Insertion site | Operon context | Fold change ^a^ | Protein description |
| --- | --- | --- | --- | --- | --- |
| M1 | *clpP* | 2964736 | *tig-clpP* | 4.5 | ATP-dependent Clp protease adaptor protein |
| M2 | *clpA* | 2964452 | *clpS-clpA* | 5.0 | ATP-binding protease component ClpA |
| M3 | *clpA* | 2962337 | *clpS-clpA* | 5.0 | ATP-binding protease component ClpA |
| M4 | *lasI* | 1559409 |  | -5.6 | Autoinducer synthesis protein LasI |
| M5 | *lasI* | 1559486 |  | -5.6 | Autoinducer synthesis protein LasI |
| M6 | *cdpR* | 2928582 |  | 4.4 | Probable transcriptional regulator |
| M7 | *PA0794* | 872747 | PA0793-PA0794 | -2.2 | Probable aconitate hydratase |
| M8 | *PA5218* | 5876556 | PA5216-PA5218 | 2.5 | Probable transcriptional regulator |
| M9 | *PA1429* | 1557478 |  | 7.2 | Probable cation-transporting P-type ATPase |
| M10 | *PA1429* | 1556055 |  | 7.2 | Probable cation-transporting P-type ATPase |
| M11 | *purM* | 1033791 | PA0943-PA0945 | 2.5 | Phosphoribosylaminoimidazole synthetase |
| M12 | *purM* | 1033800 | PA0943-PA0945 | 2.5 | Phosphoribosylaminoimidazole synthetase |
| M13 | *purM* | 1033816 | PA0943-PA0945 | 2.5 | Phosphoribosylaminoimidazole synthetase |
| M14 | *pyrD* | 3415729 |  | -2.8 | Dihydroorotate dehydrogenase |

**Table S2**. Mutated genes identified that altered the promoter activity of *cdpR* gene

^a^ Maximal ratio of expression (measured in counts per second) in the mutant compared to the wild-type PAO1. The ratio is reverted and marked with a minus sign when a decreased in expression was recorded in the mutant.
