# Supplementary material for: Structural and Molecular Mechanism of CdpR Involved in Quorum-Sensing and Bacterial Virulence in Pseudomonas aeruginosa
Source: PLoS Biol. 2016 Apr 27;14(4):e1002449. doi: 10.1371/journal.pbio.1002449 (PMC4847859; doi:10.1371/journal.pbio.1002449)
Supplement: S3 Table — (DOC) [file pbio.1002449.s013.doc]

**Table S3.** Bacterial strains and plasmids used in this study.

| **Strain or plasmid** | **Relevant characteristics** | **Source** |
| --- | --- | --- |
| ***E. coli*** |  |  |
| DH5α | *F^–^ φ80lacZ ΔM15 Δ(lacZYA-argF)U169 recA1 endA1 hsdR17(rk^–^, mk^+^)phoA supE44 thi-1 gyrA96 relA1 tonA* | Stratagene |
| BL21 star (DE3) | F^-^ *ompT hsdS*_B_ (rB^-^ m_B_^-^) *gal dcm met* (DE3) | Invitrogen |
| ***P. aeruginosa*** |  |  |
| PAO1 | Wild type | This lab |
| Δ*cdpR* | *cdpR* deletion mutant of PAO1; Gm^r^ | This study |
| Δ*pqsH* | *pqsH* deletion mutant of PAO1; Gm^r^ | This study |
| Δ*clpS*Δ*clpA* | *clpSclpA* deletion mutant of PAO1; Gm^r^ | This study |
| Δ*clpP* | *clpP* deletion mutant of PAO1; Tc^r^ | This study |
| Δ*cdpR*Δ*clpS*Δ*clpA* | *cdpR* and *clpSclpA* mutant of PAO1; Gm^r^, Tc^r^ | This study |
| Δ*cdpR*Δ*clpP* | *cdpR* and *clpP* double mutant of PAO1; Gm^r^, Tc^r^ | This study |
| Δ*cdpR*Δ*pqsH* | *cdpR* and *pqsH* double mutant of PAO1; Gm^r^, Tc^r^ | This study |
| Δ*cdpR/mini-CTX-cdpR* | Δ*cdpR* complemented strain, derived from Δ*cdpR* and Mini-CTX-*cdpR*; Gm^r^, Tc^r^ | This study |
| Δ*cdpR/p-cdpR* | Δ*cdpR* complemented strain, derived from Δ*cdpR* and pAK1900-*cdpR*; Gm^r^, Cb^r^ | This study |
| Δ*clpS*Δ*clpA /p-clpSclpA* | Δ*clpS*Δ*clpA* complemented strain, derived from Δ*clpS*Δ*clpA* and pAK1900-*clpSclpA*; Gm^r^, Cb^r^ | This study |
| Δ*clpP/p-clpP* | Δ*clpP* complemented strain, derived from Δ*clpP* and pAK1900-*clpP*; Gm^r^, Cb^r^ | This study |
| **Plasmids** |  |  |
| pBT20 | Mini-Tn*M* delivery vector, Ap^r^, Gm^r^ | [^1^](#_ENREF_1) |
| pET28a | T7 *lac* promoter-operator, N-terminal His tag, Kan^r^ | Novagen |
| pEX18Ap | *oriT*^+^ *sacB*^+^ gene replacement vector with multiple-cloning site from pUC18; Ap^r^ | [^2^](#_ENREF_2) |
| pEX18Tc | *oriT*^+^ *sacB*^+^ gene replacement vector with multiple-cloning site from pUC18; Tc^r^ | [^2^](#_ENREF_2) |
| pPS858 | Source plasmid of Gm^r^ cassette; Gm^r^, Ap^r^ | [^2^](#_ENREF_2) |
| pRK2013 | Broad-host-range helper vector; Tra^+^, Kn^r^ | [^3^](#_ENREF_3) |
| pMS402 | Expression reporter plasmid carrying the promoterless *luxCDABE* gene; Kn^r^, Tmp^r^ | [^4^](#_ENREF_4) |
| pAK1900 | *E. coli*-*P. aeruginosa* shuttle cloning vector carrying p*lac* upstream of MCS; Ap^r^, Cb^r^ | [^5^](#_ENREF_5) |
| pBT | p15A origin of replication, *lac-UV5* promoter, λ cI open reading fram; Cm^r^ | Agilent |
| pTRG | ColE1 origin of replication, lpp promoter, lac-UV5 promoter, RNAPα open reading frame; Tc^r^ | Agilent |
| pBT-LGF2 | Interaction control plasmid encoding the dimerization domain (40 amino acid residues) of the Gal4 transcriptional activator protein; Cm^r^ | Agilent |
| pTRG-GAII 1^p^ | Interaction control plasmid encoding a domain (90 amino acid residues) of the mutant form of the GaII 1 protein; Tc^r^ | Agilent |
| mini-CTX-*lacZ* | Integration plasmid; Tc^r^ | [^6^](#_ENREF_6) |
| mini-CTX-*lux* | Integration plasmid; Tc^r^ | [^6^](#_ENREF_6) |
| pET28a-*cdpR* | Protein expression construct, the entire gene of CdpR cloned in pET28a vector | This study |
| pET28a-*cdpR*^R263A^ | pET28a-*cdpR* with mutated R263 to A | This study |
| pET28a-*cdpR*^R274A^ | pET28a-*cdpR* with mutated R274 to A | This study |
| pET28a-*cdpR*^R312A/317A^ | pET28a-*cdpR* with mutated R312 to A and R317 to A | This study |
| pBT-*cdpR* | pBT plasmid containing the entire *cdpR* gene | This study |
| pTRG-*clpS* | pTRG plasmid containing the entire *clpS* gene | This study |
| pEX-*cdpR_Gm_* | *cdpR* deletion plasmid, pEX18Tc with 1975 bp upstream region, Gm^r^ cassette from pPS858 and 1242 bp downstream region of *cdpR*; Tc^r^, Gm^r^ | This study |
| pEX-*cdpR_Tc_* | *cdpR* deletion plasmid, pEX18Ap with 1975 bp upstream region, Tc^r^ cassette from mini-CTX-lacZ and 1242 bp downstream region of *cdpR*; Tc^r^ | This study |
| pEX-*pqsH* | *pqsH* deletion plasmid, pEX18Ap with 1970 bp upstream region, Gm^r^ cassette from pPS858 and 1954 bp downstream region of *pqsH*; Ap^r^, Gm^r^ | This study |
| pEX-*clpSclpA* | *clpSclpA* deletion plasmid, pEX18Ap with 1980 bp upstream region of *clpS*, Gm^r^ cassette from pPS858 and 1934 bp downstream region of *clpA*; Ap^r^, Gm^r^ | This study |
| pEX-*clpP* | *clpP* deletion plasmid, pEX18Ap with 2026 bp upstream region, Gm^r^ cassette from pPS858 and 2035 bp downstream of *clpP*; Ap^r^, Gm^r^ | This study |
| mini-CTX-*cdpR*-Flag | Expression plasmid, mini-CTX-*lacZ* containing the entire *cdpR* gene and the 3x *flag* sequence; Tc^r^ | This study |
| mini-CTX-*pqsH-*Flag | Expression plasmid, mini-CTX-*lacZ* containing the entire *pqsH* gene and the 3x *flag* sequence; Tc^r^ | This study |
| pAK-*cdpR* | pAK1900 with the entire *cdpR* gene; Ap^r^ | This study |
| pAK-*clpS/clpA* | pAK1900 with the entire *clpS/clpA* gene; Ap^r^ | This study |
| pAK-*clpP* | pAK1900 with the entire *clpP* gene; Ap^r^ | This study |
| pKD-*phzA1* | pMS402 containing *phzA1* promoter region; Kn^r^, Tmp^r^ | [^4^](#_ENREF_4) |
| pKD-*pqsH* | pMS402 containing *pqsH* promoter region; Kn^r^, Tmp^r^ | This study |
| pKD-*cdpR* | pMS402 containing *cdpR* promoter region; Kn^r^, Tmp^r^ | This study |
| CTX-*cdpR-lux* | Integration plasmid, CTX6.1 with a fragment of pKD-*cdpR*; Tc^r^ | This study |
| CTX-*phzA1-lux* | Integration plasmid, CTX6.1 with a fragment of pKD-*phzA1*; Kn^r^, Tc^r^ | [^7^](#_ENREF_7) |
| CTX-*pqsH-lux* | Integration plasmid, CTX6.1 with a fragment of pKD-*pqsH*; Kn^r^, Tc^r^ | This study |
| CTX-*clpP* | Integration plasmid, Mini-CTX-*lux* with the entire *clpP* gene; Tc^r^ | This study |
| CTX-*clpS/clpA* | Integration plasmid, Mini-CTX-*lux* with the entire *clpS/clpA* gene; Tc^r^ | This study |

**Reference**

1. Kulasekara, H.D. *et al.* A novel two-component system controls the expression of *Pseudomonas aeruginosa* fimbrial cup genes. *Mol Microbiol* **55**, 368-80 (2005).

2. Hoang, T.T., Karkhoff-Schweizer, R.R., Kutchma, A.J. & Schweizer, H.P. A broad-host-range Flp-FRT recombination system for site-specific excision of chromosomally-located DNA sequences: application for isolation of unmarked *Pseudomonas aeruginosa* mutants. *Gene* **212**, 77-86 (1998).

3. Ditta, G., Stanfield, S., Corbin, D. & Helinski, D.R. Broad host range DNA cloning system for gram-negative bacteria: construction of a gene bank of *Rhizobium meliloti*. *Proc Natl Acad Sci U S A* **77**, 7347-51 (1980).

4. Duan, K., Dammel, C., Stein, J., Rabin, H. & Surette, M.G. Modulation of *Pseudomonas aeruginosa* gene expression by host microflora through interspecies communication. *Mol Microbiol* **50**, 1477-91 (2003).

5. Poole, K., Neshat, S., Krebes, K. & Heinrichs, D.E. Cloning and nucleotide sequence analysis of the ferripyoverdine receptor gene *fpvA* of *Pseudomonas aeruginosa*. *J Bacteriol* **175**, 4597-604 (1993).

6. Becher, A. & Schweizer, H.P. Integration-proficient *Pseudomonas aeruginosa* vectors for isolation of single-copy chromosomal *lacZ* and *lux* gene fusions. *BioTechniques* **29**, 948-953 (2000).

7. Liang, H., Duan, J., Sibley, C.D., Surette, M.G. & Duan, K. Identification of mutants with altered phenazine production in *Pseudomonas aeruginosa*. *J Med Microbiol* **60**, 22-34 (2011).
