# Supplementary material for: Structural and Molecular Mechanism of CdpR Involved in Quorum-Sensing and Bacterial Virulence in Pseudomonas aeruginosa
Source: PLoS Biol. 2016 Apr 27;14(4):e1002449. doi: 10.1371/journal.pbio.1002449 (PMC4847859; doi:10.1371/journal.pbio.1002449)
Supplement: S4 Table — (DOCX) [file pbio.1002449.s014.docx]

**Table S4**. Primers used in this study.

| Primer | Sequence (5'→3')^a^ | Application |
| --- | --- | --- |
| *cdpR*-VSV-F | AGCAAGCTTCCCCTTACGCAAGGTAGTG | CdpR ChIP-seq |
| *cdpR*-VSV-R | GTTTCTAGAGCGTTCGCCCTGGCGCCG |  |
| pET-*cdpR*-S | TGCGGATCCATGAGCCCGTCCGAAAACAT | Protein cloning |
| pET-*cdpR*-A | GTCAAGCTTGCGTTCGCCCTGGCGCCGCG |  |
| pBT-*cdpR*-S | GTAgcggccgcaATGAGCCCGTCCGAAAACAT | Bacterial two-hybrid |
| pBT-*cdpR*-A | GATgaattcCTAGCGTTCGCCCTGGCGCC |  |
| pTRG-*clpS*-S | GTAgcggccgcaGTGGTACTGTTCAACGACG | Bacterial two-hybrid |
| pTRG-*clpS*-A | GATgaattcTCAACTGTCTTTCTCTATCT |  |
| pEX-*cdpR*-up-S | GTAggatccACATCATTCGACGGCATC | Constructing *cdpR* deletion mutant |
| pEX-*cdpR*-up-A | GCGtctagaTTACCTCTTGCACGCGAT |  |
| pEX-*cdpR*-down-S | GCGtctagaGCCAACGACTATCGCCAG |  |
| pEX-*cdpR*-down-A | GCGaagcttGTGGTGGCGATCATGCTC |  |
| pEX-*clpS*-up-S | GTAggatccAAAAGATCCAGGTGCC | Constructing *clpS/clpA* deletion mutant |
| pEX-*clpS*-up-A | GCGtctagaGGCGTGTAATCGTCGTTG |  |
| pEX-*clpA*-down-S | GCGtctagaTCGAGTTCGAGATCACGG |  |
| pEX-*clpA*-down-A | GGCaagcttCTCGACGACCATTGATCG |  |
| pEX-*clpP*-up-S | GCTGAATTCGTTCAGCTTGCCGAATGTCA | Constructing *clpP* deletion mutant |
| pEX-*clpP*-up-A | TGCTCTAGAAGCCTCAAAGACGCACAAG |  |
| pEX-*clpP*-down-S | GCGTCTAGATACTGGCGGTCGAGACATT |  |
| pEX-*clpP*-down-A | TGTAAGCTTCTGCTCGATCTTCAGCG |  |
| pEX-*pqsH*-up-S | GCGaagcttCTCGGCATGGCCTGGTTC | Constructing *pqsH* deletion mutant |
| pEX-*pqsH*-up-A | GCGtctagaGGCATCCGTCGCGACTTC |  |
| pEX-*pqsH*-down-S | GCGtctagaTTGGTCGCCTGTTTCGAC |  |
| pEX-*pqsH*-down-A | GCGgaattcAACCAGCACCGGGAAATC |  |
| pAK-*cdpR*-S | AGCAAGCTTCCCCTTACGCAAGGTAGTG | Constructing *cdpR* complemented plasmid |
| pAK-*cdpR*-A | GTTTCTAGAGCGTTCGCCCTGGCGCCG |  |
| pAK-*clpA*-S | GCTtctagaCCAGCATCCATTGCTCTGC | Constructing *clpA* complemented plasmid |
| pAK-*clpA*-A | GTCaagcttAGTCGACACGAGCCGCTG |  |
| pAK-*clpS*-S | GCTtctagaTCAGGACCATCCCGAGCC | Constructing *clpS* complemented plasmid |
| pAK-*clpS*-A | GTCaagcttCAACATGGCTTCACCTCGC |  |
| pAK-*clpP*-S | ACTggatccCACTGTGACCGATAAGCA | Constructing *clpP* complemented plasmid |
| pAK-*clpP*-A | ACGaagcttGATCCAATGGGTAGCTTC |  |
| Mini-*cdpR*-flag-S | TACgtcgacCGATGGCGCCTGAAGCTC | Western-blot |
| Mini-*cdpR*-flag-A | ACTaagcttGCGTTCGCCCTGGCGCCG |  |
| Mini-*pqsH*-flag-S | TATgtcgacTGGCTGCGCCTGGATGATCG | Western-blot |
| Mini-*pqsH*-flag-A | GTCaagcttCTGTGCGGCCATCTCACCGA |  |
| *cdpR*-*lux*-F | GTActcgagACGCGTGGCCGGAAATCC | Constructing *cdpR* promoter plasmid |
| *cdpR*-*lux*-R | GTAggatccATGCGTTGCACGGCGAGG |  |
| *pqsH*-*gf* | TAAGGGGTTGACAGGAGCG | For gel-shift |
| *pqsH*-*gr* | GACCAGCAGCCAGTCGAT |  |
| *pqsH-p1-gf* | CGTCGTGGGCGGTGGTGGC | For gel-shift |
| *cerN*-gf | CTACCTGGCGCTGCTGAT | For gel-shift |
| *cerN*-gr | CCTGGCAGGCATGGAGAG |  |
| *sphR*-gf | ATCCGAGCTGATCTTCACGT | For gel-shift |
| *sphR*-gr | GTGGATACCTGGGCGAGTT |  |
| *opdC-gf* | ACTACATCAACCAGGGCGAC | For gel-shift |
| *opdC-gr* | ACGTCGATGGGATAGTCGAC |  |
| *PA0440-gf* | CGTACGCAACAACGCCCAGG | For gel-shift |
| *PA0440-gr* | CCGAGGAACACCGCGTCGTA |  |
| *PA1271-gf* | ACCAGGTCTTCGGCAACTCC | For gel-shift |
| *PA1271-gr* | CCGAACTGCTGGTTGTCGTC |  |
| *pscC-gf* | GCGGCGGCCTGCTTGAAATC | For gel-shift |
| *pscC-gr* | ACCAGGTGGACTGCCGCGAT |  |
| *cysG-gf* | GCCTGCTGGCCGACGCCGGC | For gel-shift |
| *cysG-gr* | GCGGCTGGCGAGTCCGGCCA |  |
| *PA3388-gf* | CGACCAGGTGGAAGCGCTGG | For gel-shift |
| *PA3388-gr* | CTGGCCGTGCTCATGGGCCT |  |
| *PA4087-gf* | CTGACCGGCGTGGAGGCCGA | For gel-shift |
| *PA4087-gr* | CAGGGCGCCGACCGGGAAGT |  |
| *recC-gf* | ACCGATGCCAGAACCCTTGG | For gel-shift |
| *recC-gr* | AACTCGGGACGCTCCAGCAA |  |
| *PA4772-gf* | TGCTGCTCGTCCTCGGCGAC | For gel-shift |
| *PA4772-gr* | GGCGGATCTTCGCGGCCAGT |  |
| *PA5146-gf* | CTGAACTTCACCAACGGCGT | For gel-shift |
| *PA5146-gr* | CCTTCAGGTGTCATTTGCGG |  |
| *PA0159-gf* | CGGTGGACGACCTGGAGCGC | For gel-shift |
| *PA0159-gr* | GTCGCGGGCGAATGCCTGGG |  |
| *serS-gf* | CCACCCGTGGCTTCCAACTG | For gel-shift |
| *serS-gr* | TCACCGAGGGCGACATGGTC |  |
| *valS-gf* | GTCGAAGGCTTCCGCAACTT | For gel-shift |
| *valS-gr* | GTGATGAACGGCATGAACGG |  |
| *PA3992-gf* | CGAATACAACCAGCCTGCCG | For gel-shift |
| *PA3992-gr* | CATCCGCATCCACCGCATAG |  |
| *PA4513-gf* | TCGTGGTCAGCACCTTCGGC | For gel-shift |
| *PA4513-gr* | AGGATTCAGGCACTCCCGCC |  |
| *PA4541-gf* | TGTACGGTGGGCTCATCGGC | For gel-shift |
| *PA4541-gr* | TCTCCGAGCAGACCGCCAAC |  |
| *selB-gf* | CTCGGCCCCCGGTTGTTCGC | For gel-shift |
| *selB-gr* | CATCCTGGCGCAGCTCGCGG |  |
| *PA5114-gf* | CCGAGCGCGGCGAACTGCTG | For gel-shift |
| *PA5114-gr* | GCGGCGTTGCTGGTGCAGGC |  |
| Tc-S | TTTTCTAGACGGTCGCTACCATTACCAGT | Amplifying Tc^r^ cassette |
| Tc-A | TTTTCTAGACTGGTGAGTCAAGGGTTGGT‍ |  |
| CdpR^R263A^-F | TCACCGAGCGGACGCTGGCCCGACGGCTCGCCGA | Mutagenesis |
| CdpR^R263A^-R | TCGGCGAGCCGTCGGGCCAGCGTCCGCTCGGTGA |  |
| CdpR^R274A^-F | AGGGCACCAACTACGCGGAAGTGCTCGACCTGGT | Mutagenesis |
| CdpR^R274A^-R | ACCAGGTCGAGCACTTCCGCGTAGTTGGTGCCCT |  |
| CdpR^R312A/R317A^-F | GTTTTGCCCACGCCTTCCGTGCCTGGACGGGACA | Mutagenesis |
| CdpR^R312A/R317A^-R | TGTCCCGTCCAGGCACGGAAGGCGTGGGCAAAAC |  |

^a^ Restriction sites are underlined.
