# Supplementary material for: Structural and Molecular Mechanism of CdpR Involved in Quorum-Sensing and Bacterial Virulence in Pseudomonas aeruginosa
Source: PLoS Biol. 2016 Apr 27;14(4):e1002449. doi: 10.1371/journal.pbio.1002449 (PMC4847859; doi:10.1371/journal.pbio.1002449)
Supplement: S5 Table — (DOCX) [file pbio.1002449.s015.docx]

**Table S5**. Data collection and structural refinement statistics

| Se-*Pa*CdpR (PDB ID: 5CHH) | |
| --- | --- |
| **Date collection** | |
| Space group | P4_1_2_1_2 |
| Resolution range (Å) ^a^ | 30.0-1.85 (1.92-1.85) |
| Unit cell parameters | 109.8 109.8 66.1 90 90 90 |
| Unique reflections ^a^ | 35195 (3459) |
| Completeness (%)^a^ | 99.9 (99.8) |
| R_merge_ (%)^a^ | 6.8 (44.2) |
| I/s(I) ^a^ | 28.7 (2.0) |
| Redundancy ^a^ | 10.0 (7.1) |
| **Refinement** | |
| Rwork (%)^a, b^ | 18.8 (27.1) |
| R_free_ (%)^a, c^ | 23.4 (34.3) |
| Number of atoms  Protein  water | 2556  88 |
| Overall B-factors (Å^2^) | 39.8 |
| RMSD  bond lengths (Å )  bond angles (°) | 0.005  0.963 |
| Ramachandran plot, residues in (%)  Most favored region  Allowed region  Disallowed region | 97.8  2.2  0 |

^a^Values in parentheses are for the highest resolution shell.

^b^Rwork = Σ ||F_obs_| − |F_calc_||/Σ |F_obs_|, where F_obs_ and F_calc_ are the observed and calculated structure factors, respectively.

^c^R_free_ = Σ ||F_obs_| − |F_calc_||/Σ |F_obs_| for 5% of the data not used during structural refinement.
